# Long-term severe hypoxia adaptation induces non-canonical EMT and a novel Wilms Tumor 1 (WT1) isoform

**DOI:** 10.1038/s41417-024-00795-3

**Published:** 2024-07-08

**Authors:** Jordan Quenneville, Albert Feghaly, Margaux Tual, Kiersten Thomas, François Major, Etienne Gagnon

**Affiliations:** 1 Institute for Research in Immunology and Cancer, Université de Montréal, Montréal, QC Canada; 2 Department of Molecular Biology, Université de Montréal, Montréal, QC Canada; 3 Department of Microbiology, Infectiology, and Immunology, Faculty of Medicine, Université de Montréal, Montréal, QC Canada; 4 Department of Integrative Oncology, BC Cancer Research Center, Vancouver, BC Canada; 5 Department of Computer Science and Operations Research, Faculty of Arts and Sciences, Université de Montréal, Montréal, QC Canada

**Keywords:** Gene expression analysis, Cancer microenvironment, Cancer models, Melanoma, Ovarian cancer

## Abstract

The majority of cancer deaths are caused by solid tumors, where the four most prevalent cancers (breast, lung, colorectal and prostate) account for more than 60% of all cases (1). Tumor cell heterogeneity driven by variable cancer microenvironments, such as hypoxia, is a key determinant of therapeutic outcome. We developed a novel culture protocol, termed the Long-Term Hypoxia (LTHY) time course, to recapitulate the gradual development of severe hypoxia seen in vivo to mimic conditions observed in primary tumors. Cells subjected to LTHY underwent a non-canonical epithelial to mesenchymal transition (EMT) based on miRNA and mRNA signatures as well as displayed EMT-like morphological changes. Concomitant to this, we report production of a novel truncated isoform of WT1 transcription factor (tWt1), a non-canonical EMT driver, with expression driven by a yet undescribed intronic promoter through hypoxia-responsive elements (HREs). We further demonstrated that tWt1 initiates translation from an intron-derived start codon, retains proper subcellular localization and DNA binding. A similar tWt1 is also expressed in LTHY-cultured human cancer cell lines as well as primary cancers and predicts long-term patient survival. Our study not only demonstrates the importance of culture conditions that better mimic those observed in primary cancers, especially with regards to hypoxia, but also identifies a novel isoform of WT1 which correlates with poor long-term survival in ovarian cancer.

## Introduction

Approximately 1 in 3 deaths in industrialized countries are caused by cancer, with the majority of deaths arising from solid tumors [1]. The most prevalent solid cancers account for almost half of all cancers in highly developed countries [2]. It has become clear that effective therapy must address tumor cell heterogeneity and the microenvironment [3]. Intratumoral areas contain high physiological variability in nutrients, pH, and oxygen availability leading to tumor cell heterogeneity [4, 5]. Low oxygen availability (hypoxia) is particularly deleterious to patient survival as it renders tumor cells more resistant to chemotherapy, radiotherapy, and immunotherapy [5]. Resistance to treatment can be due to both the characteristics of the hypoxic microenvironment and intrinsic cancer cell features [5]. To survive hypoxic conditions, tumor cells adapt through the Hypoxia Induced Factors (HIFs), which promote phenotypes including but not limited to cell survival, motility, angiogenesis, and altered glucose metabolism. As a result, hypoxia adaptation is regarded as a fundamental force driving tumor cell pathogenesis [5]. Therefore, an accurate understanding of the breadth of hypoxic adaptations and consequences is essential to the development of more effective therapeutics.

Although hypoxia adaptation is primarily orchestrated by HIF1α, HIF2α has also been shown to play an important role [5]. Both proteins are regulated through oxygen-dependent pathways triggered by proline hydroxylation leading to proteasomal degradation under normal oxygen conditions (normoxia) [5]. Under hypoxic conditions (<5% O_2_), both HIF1α and HIF2α escape degradation, translocate to the nucleus, and associate with HIF1β/ARNT to form the functional transcription factors HIF1 and HIF2, respectively, and initiate transcription [5]. HIF1 drives the transcription of hundreds of mRNAs and miRNAs that enable cell adaptation to and beyond hypoxia such as genes linked to metastasis through the induction of epithelial to mesenchymal transition (EMT), which can occur through canonical and non-canonical pathways [6, 7]. Tumor cells which can initiate EMT dramatically decrease survival probabilities in cancer patients [8]. Thus, hypoxia and the HIF1 transcriptional program are potent microenvironmental and cellular forces, pushing tumor cells towards a more pathogenic and metastatic cell state.

When studying hypoxia adaptation in vitro, most culture protocols abruptly transition cells from atmospheric oxygen levels (21% O_2_) to hypoxic conditions (1% O_2_ or below) [9]. However, during tumor development, hypoxic development begins at physoxia (normal tissue oxygenation) and develops over a longer time scale as the tumor and vasculature grow erratically [4]. In addition, most culture protocols do not reach severe hypoxic and anoxic (an absence of oxygen) levels characteristic of established tumor microenvironment [4]. Recent studies employing sustained hypoxic cell culture have highlighted the fact that hypoxic culture conditions greatly affect tumor cell adaptation and are more representative of observations made in vivo [10–13].

Here, we report the development and characterization of a novel in vitro hypoxia adaptation protocol designed to mimic the gradual development of the severely hypoxic microenvironment observed in vivo. Cells subjected to this protocol undergo a non-canonical EMT spontaneously and produce a novel truncated isoform of WT1 transcription factor (tWt1), a known oncogene and EMT promoter [14]. Induction of EMT and tWt1 were both dependent on hypoxia severity and adaptation time. Finally, molecular characterization of the novel tWt1 isoform suggests a limited but active functionality, and its nearest human ortholog correlates with poor long-term survival.

## Materials and methods

### Cell lines and cell culture

#### WT Cell lines

B16-F10 (CRL-6475) and HEK293T (CRL-3216) cells were obtained from ATCC. MEL1300, SK-MEL23, MEL537 were provided by Dr. Réjean Lapointe at the Centre de Recherche du Centre Hospitalier de l’Université de Montréal (CRCHUM). All derived cell lines described in this study were obtained through stable transduction cells with various lentiviruses encoding for the gene or construct of interest (described below). ZR75 cells were provided by the lab of Dr. Sylvie Mader at l’Institut de Recherche en Immunologie et Cancérologie (IRIC). OVCAR3 cells were provided by the lab of Dr. Clause Perrault at IRIC. TOV3291G was provided by the lab of Dr. Anne-Marie Mes-Masson at the CRCHUM. All cell lines were routinely tested for the presence of mycoplasma.

#### B16-HG cells

Human HIF1α, obtained from HEK293T cell cDNA, was cloned as a GFP fusion protein, and inserted into the pHAGE lentiviral backbone using Gibson assembly. To enable protein expression of this fusion protein, a 10 amino acids long GS linker replaced the HIF1α stop codon upstream of the GFP. After validating the construct through sequencing, lentiviruses were made and used to transduce B16-F10 cells at an MOI of < 0.3 to maximize single integration. The B16-HG cell line was then generated through FACS single-cell sorting after CoCl_2_-mediated stabilization of HIF1α to obtain a pure population.

#### Truncated WT1-GFP expressing cell lines

The murine E6 and E7 isoforms of WT1 were amplified by PCR from cDNA obtained from the B16-HG cell line at the 0.1% O_2_ condition following the LTHY protocol. The human G and P-tWT1 isoforms were amplified from OVCAR3 cDNA. All isoforms were cloned into a doxycycline (Dox) inducible lentiviral backbone (pCW) at the N-terminus of an SGSGS linker and ATG-deficient GFP via Gibson assembly. The pCW lentiviral construct also contained a puromycin selection gene driven by a separate promoter, along with rtTA. The KTS motif was later removed from the murine tWt1 construct via Gibson assembly. The murine tWT1-GFP isoforms were expressed in B16-F10 melanoma cells through stable transduction in normoxia. The human tWT1-GFP isoforms were expressed in HEK293T cells in normoxia. The murine crit-tWt1 construct was generated from the E7-WT1 CDS through Gibson assembly using a unique F primer (GTAAAGTCGAGCTTGCGTTGCTAGCCACCATGAAGACCCACACCAGGAC).

#### Normoxic cell culture

B16-F10 and its derived cells lines, 293T cells, all human melanoma cell lines, and OVCAR3 cells were maintained in DMEM + GlutaMax (ThermoFisher: 10569-010) supplemented with 10% FBS (Wisent Bioproducts: 090150) & 1% Penicillin-Streptomycin (Wisent Bioproducts: 450-201-EL). Under normal tissue culture conditions, ZR75 cells were cultured in RPMI-1640 (Wisent) supplemented with 10% FBS, 100 ng/ml of Pen/Strep, 10 mM of HEPES, and 1 mM of sodium pyruvate. During LTHY, ZR75 cells were cultured in phenol red-free DMEM (Wisent 319-065 CL) supplemented with 10% charcoal-stripped FBS, 4 mM of L-glutamine, 100 ng/ml of Pen/Strep, with or without 25 nM of estradiol (E2). TOV3291G cells were maintained in OSE (Wisent: 316-030 CL) media supplemented with 10% FBS and 1% Penicillin-Strepomycin. All cells were confirmed to by mycoplasma negative at the time of experiment. For passaging, cells were detached using PBS, 10 mM HEPES pH 7.6, 0.5% FBS, and 2 mM EDTA.

#### Long term hypoxia (LTHY) incubation

All hypoxic incubations were performed in a BioSpherix Xvivo system model X2 closed hypoxic incubation system. Maintenance, calibration and general cell culture conditions were performed according to manufacturer’s recommendations. During LTHY experiments, O_2_ was controlled and maintained at set levels during the length of the time course as indicated, while CO_2_ was consistently maintained at 5%. To minimize variations in culture media oxygen levels throughout hypoxic experiments, culture media was kept within the hypoxia system at the same culture conditions as the incubator. Starting from the 0.5% O_2_ time point, media was changed every 24 h to avoid excessive media acidification due to glycolysis and lactate production. To minimize reoxygenation of cells during microscopic observations or other outer hypoxia chamber manipulations, cells were cultured in plug seal flasks (VWR, cat: 82051-070).

### Bioinformatics analyses

#### GSEA analyses

GSEA was run locally for all analyses GSEA for Linux v4.2.2. Using an in-house Python script, for each DESeq2 comparison, the gene expression table was filtered to genes with a FC > = 1.2 or FC < = 1/1.2 and a padj value < 0.05. Normalized DESeq2 expression values and the OGS for genes passing these filters were saved to a new GSEA-compatible format. GSEA was run using the log2 ratio of classes metric, the weighted scoring scheme, the gene set permutation mode, 1000 gene set permutations, and the GSEA mouse gene symbol to human ortholog file v7.5.1.

#### Heatmap generation

MiRs were filtered by a padj < 0.05 when comparing 5% vs 0.1% O_2_, and minimal expression >= 100 mean DESeq2 normalized reads in any condition comparison using Python. The heatmap was generated in R using the pheatmap library (v1.0.8). Gene expression was normalized using the contribution metric. Essentially, the miR expression at each timepoint is converted to a percentage of the total expression for that miR. The cluster separation method was done with the pheatmap argument cutree_row, with the number of clusters chosen subjectively.

For the DEG heatmap, the same statistical and expression thresholds as the miRs were used. Gene expression was normalized using the row z-score metric. Normalized gene expression patterns were clustered using kmeans in R. The number of clusters was chosen to be seven based on the elbow method and the within-group sum of squared distances. Heatmap gaps indicate separate kmean clusters. Rendering was done in R using the pheatmap library (1.0.8).

### Gene expression profile generation

Gene/miR expression histograms were generated using normalized replicate expression values from DESeq2. Histograms were rendered using GraphPad Prism7. Statistical analyses were performed in DESeq2.

Additional methodology details can be found in the supplemental materials.

## Results

### Long-term hypoxia adaptation leads to EMT-like morphological changes

**Fig. 1:**
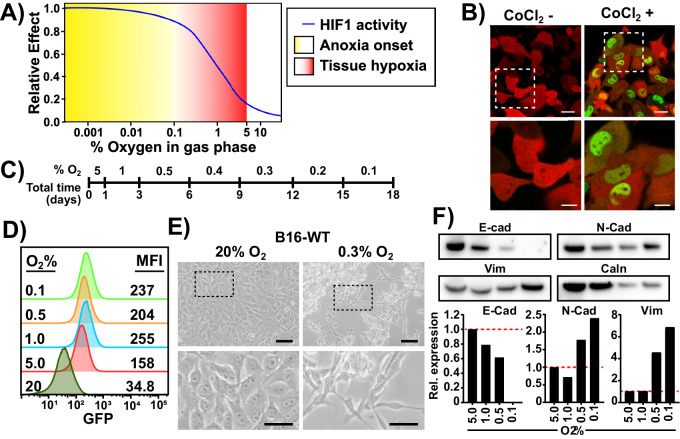
Long-term severe hypoxia adaptation induces EMT-like morphological changes. **A** The definitions of hypoxia and anoxia commonly used in the literature and their effect on HIF activity. **B** Confocal microscopy images of B16-HG cells under normal culture conditions (left), or after CoCl_2_ treatment (200 µM, 24 h). Scale bars: top = 20 µm; bottom = 10 µm. Green: HIF1α-GFP. Red: mCherry (cytoplasmic). **C** Definition of the Long-Term Hypoxia (LTHY) time course. **D** GFP levels of B16-HG cells over the course of the LTHY protocol. Numbers represent geometric Mean Fluorescent Intensity (geoMFI) of GFP signal. **E** B16-HG cell morphology under normal tissue culture conditions (left). B16-HG cell morphology after reaching the 0.3%O_2_ timepoint of the LTHY protocol (right). Scale bars: top = 100 µm; bottom = 50 µm. **F** Western blot (top) and analysis (bottom) of B16-HG harvested at indicated LTHY time points and probed for EMT-associated E-Cadherin (E-Cad), N-cadherin (N-Cad) and vimentin (Vim) and loading control calnexin (Caln). Band intensities were normalized to loading control. Data expressed as expression fold change relative to the 5%O_2_ condition.

The intratumoral microenvironment develops hypoxia over an extended time frame, generating a continuum of oxygen concentrations, resulting in differential HIF1 activity (Fig. 1A) [15]. First, to monitor tumor cell adaptation to long term and severe hypoxia, we established a reporter cell line using a lentiviral HIF1α-eGFP fusion construct in B16-F10 mouse melanoma cells (Fig. S1A). Flow cytometry and confocal microcopy analyses revealed that most cells (98%+) transduced with this construct had no GFP signal under normoxia, but had clear nuclear accumulation of GFP under HIF1α stabilizing conditions (Fig. 1B, Fig. S1B, C). We single-cell sorted GFP positive cells following CoCl_2_ treatment to obtain a clone, termed B16-HG, with high HIF1α-eGFP accumulation while remaining GFP negative under normoxic conditions and confirmed integrity of the protein fusion product by Western blot (Fig. S1C-E). Finally, we monitored the dynamics and longevity of B16-HG GFP signal following incubation in severe hypoxia and hypoxia recovery to determine the sensitivity and precision of ascribing GFP signals to a cell hypoxic state. B16-HG cells transferred directly from normoxia (TC) to 0.2% O_2_ expressed detectable GFP signal in as little as 2 h, peaked at 14 h and remained stable for a minimum of 5 h after re-oxygenation with full loss of GFP signal observed after 24 h (Fig. S1F, G). Other than a small but statistically significant difference in proliferation profile compared to the polyclonal B16-WT counterpart, most likely due to the high expression of mCherry and HIF1α-GFP mRNA, the B16-HG phenotype remained unaltered, (Fig. S1H). Nevertheless, based on these results, we determined that the B16-HG cell line can accurately track hypoxia-adaptation.

To recapitulate the gradual onset and near anoxic tumor microenvironments, we developed a long-term hypoxia (LTHY) incubation protocol, where cells endure increasingly severe hypoxia after days of acclimatization (Fig. 1C). These kinetics mimic the overall time course of tumor progression in the B16 mouse melanoma model and oxygen levels previously described in melanoma [16–18]. Flow cytometry analyses of B16-HG cells during LTHY adaptation revealed partial stabilization of HIF1α-GFP at physoxia (5% O_2_) and reached maximum stabilization at 1% O_2_ and below (Fig. 1D) [19].

Interestingly, we observed significant morphological changes in B16-HG cells during LTHY (Fig. 1E). Starting from an epithelial-like “cobblestone” morphology in normoxic to mild hypoxic conditions (5% and 1% O_2_), the cells developed a more mesenchymal-like morphology, with elongated and polarized cell features under more severe hypoxic conditions (below 0.5% O_2_), formed large aggregates, and were semi-attached to the culture dish. These morphological changes, in addition to increased expression of Vimentin (Vim) and N-cadherin (N-Cad), and decreased expression of E-cadherin (E-cad) made us investigate whether the cells were undergoing EMT (Fig. 1F).

### LTHY adaptation upregulates EMT-promoting miRs

**Fig. 2:**
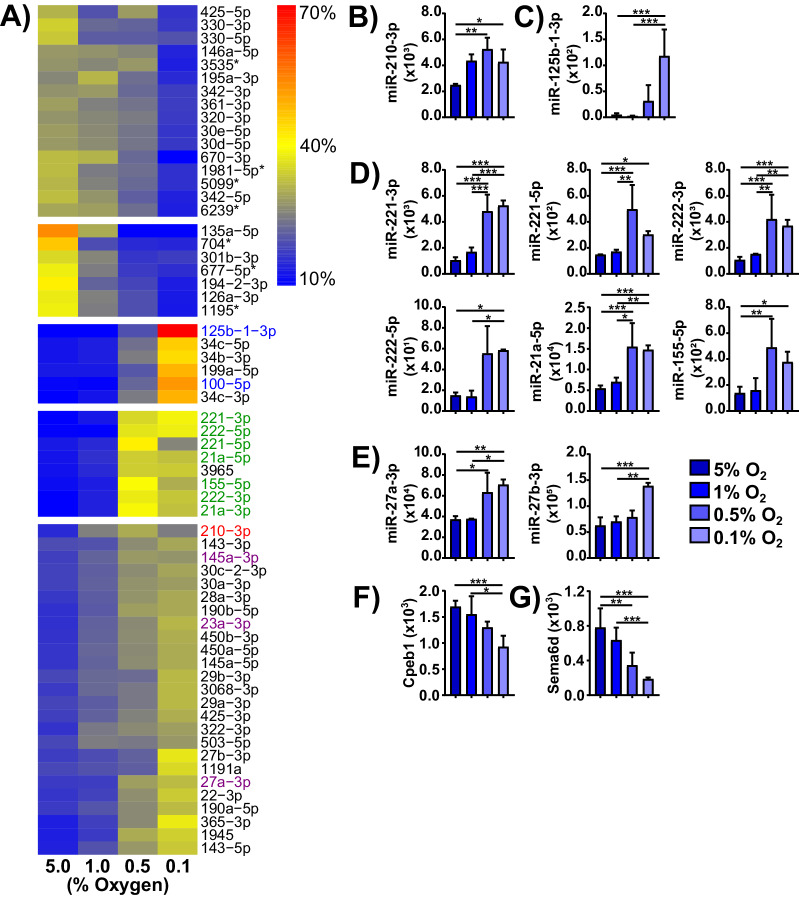
Long-term hypoxia adaptation induces an miRNA signature linked to EMT. **A** Hierarchically clustered heatmap of all differentially expressed (Benjamini-Hochberg adjusted *p*-value < 0.05 in the 5% vs 0.1% O_2_ comparison, and > 100 normalized DESeq2 reads in any condition) miRs, normalized to contribution to total expression in the dataset. Red denotes the classical hypoxia-induced miR, miR-210-3p. Green denotes EMT-promoting miRs miR-221/222. Blue and purple denote other miRs of interest. Heatmap clustered using WardD.2 hierarchical linkage metric, with the number of clusters chosen subjectively. **B** Expression values for miR-210-3p. **C** Expression values for miR-125b-1-3p. **D** Expression values for miRs of interest in cluster 4. **E** Expression values for miRs of interest in cluster 5. **F**, **G** Expression values for genes Cpeb1 and Sema6d. **B**, **G** Expression levels are DESeq2 normalized reads. * denotes relative significance as calculated by DESeq2 Benjamini-Hochberg adjusted *p*-value (padj). *padj < 0.05, **padj < 0.01, ***padj < 0.001.

Given these observations, we investigated whether cells were indeed undergoing EMT at the transcriptomic level. We collected miRNAseq and mRNAseq data at end of the 5%, 1%, 0.5%, and 0.1% O_2_ time points during LTHY to identify differentially expressed miRs (DEmiRs) and genes (DEGs). PCA analyses confirmed that oxygen content was a major determinant in shaping the transcriptome, as PC1 correlates with the LTHY stages (Fig. S2A). After verifying the expression of all components of the miRNA biogenesis and effector pathways during LTHY (Fig. S2B), we performed hierarchical clustering analyses, which revealed a dynamic DEmiR landscape across conditions with two clusters (clusters 3&4) being highly regulated at the 1-0.5% and 0.5-0.1% O_2_ transitions. (Fig. 2A). As a validation step, we examined miR-210-3p, a canonical hypoxia-induced miRNA. Although miR-210-3p was already elevated at 5% O_2_, indicative of some level of hypoxic stress, levels were further significantly upregulated during LTHY, indicative of an increased state of hypoxic stress (Fig. 2A, B). These results agree with our previous observations made with HIF1α-GFP (Fig. 1D).

Interestingly the top DEmiR at 0.1% O_2_ was miR-125b-1-3p, which has been linked to increased metastatic potential in colorectal cancer cells and was the top upregulated miR in an EMT-inducing assay using pancreatic cancer cells along with miR-100-5p (Fig. 2A, C) [20, 21]. All other miRs present in this cluster also have links to EMT and have been shown to be regulators of TGFβ-induced EMT [22–25]. Remarkably, except for the uncharacterized miR-3965, all of the miRs that were significantly upregulated at 0.5% O_2_, concomitant with observed morphological changes, are known to be positively correlated or directly involved in EMT (Fig. 2D) [26–35]. Most notably, miR-221 and miRs-222 are directly involved in EMT [26, 27, 36–38].

Furthermore, other miRNAs with direct links to EMT were identified as significantly increased during LTHY (Fig. 2A, E) [39, 32]. Indeed, miR-145a-3p and miR-23a-3p, shown to promote EMT through the repression of CPEB1 and SEMAD6 respectively, were upregulated during LTHY which correlated with a reduction of their targets (Fig. 2F, G) [40, 41]. We observed a similar trend for miR-27a/b, another EMT inducing miR, although the impact on their known targets was less pronounced (Fig. S2C, D) [32–35, 39]. Other miRs in this cluster have all been linked to EMT [42–44]. Complimentarily, except for just few (identified with asterisk), all miRNAs downregulated in LTHY are known oncogenic, motility/invasion or EMT suppressors providing an even stronger case for LTHY-induced EMT. Of these, miR-146a-5p and miR-330 have been shown to promote apoptosis and reduce levels of Vim [45, 46]. Together, miRNA profiling across LTHY supports the hypothesis that spontaneous EMT occurs at 0.5% O_2_, but that the pathways leading to EMT may differ from canonical pathways. It should be noted that all miRNAs identified within these clusters share a high degree of sequence homology or the exact sequence to their human orthologs, which suggests a conservation of function, as is the case for microRNA regulatory circuits [47].

### LTHY adaptation induces non-canonical EMT

**Fig. 3:**
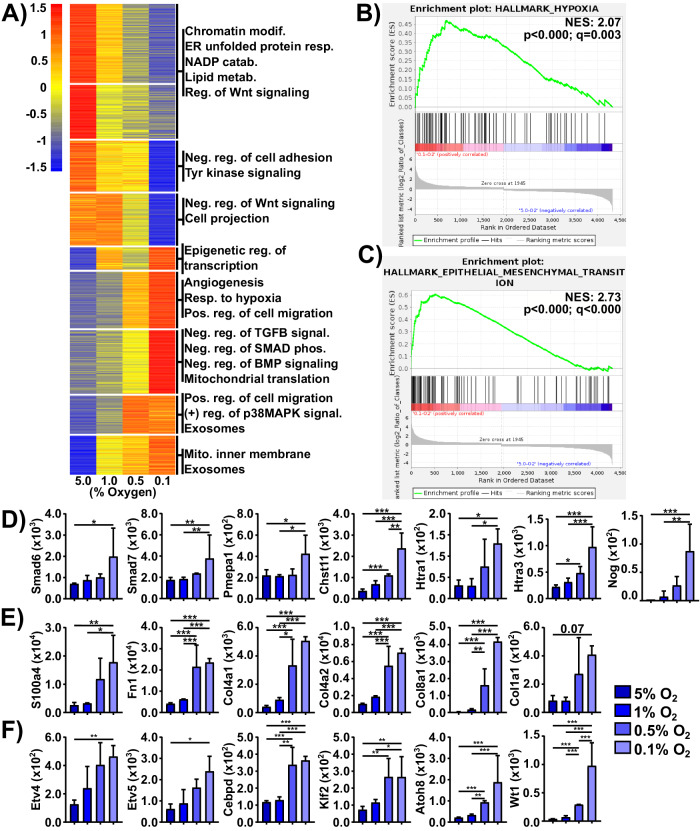
Long-term hypoxia adaptation induces non-canonical EMT at the mRNA level. **A** LTHY DEG k-means clustered heatmap. Gene expression normalized using row Z-score. Cluster number was determined using the elbow method. GO term enrichment was done using DAVID and cluster gene lists as input. Displayed GO terms are all significantly enriched (*p* < 0.05). **B**, **C** GSEA of LTHY 5% vs 0.1% DEGS. **B** Enrichment plot for Hallmark of Hypoxia. **C** Enrichment plot for Hallmark of Epithelial-Mesenchymal Transition. Normalized Enrichment Scores (NES) and statistical significance are found in each plot, as calculated by GSEA. **D** Expression values for genes associated with negative regulation of TGFβ and BMP signaling, and negative regulation of SMAD phosphorylation. **E** Expressions of EMT effector genes. **F** Expressions of potential EMT-driving genes. **D**–**F** Values are DESeq2 normalized reads, error bars are SD. * denotes relative significance as calculated by DESeq2 Benjamini–Hochberg adjusted *p*-value (padj). *padj < 0.05, **padj < 0.01, ***padj < 0.001.

To further investigate the LTHY-induced EMT-like state across hypoxic conditions at the mRNA level, we performed non-hierarchical clustering of DEGs followed by Gene Ontology (GO) term analyses to identify DEGs and assess potential functional pathways (Fig. 3A). Statistically significant enrichment for the EMT-associated phenotype “positive regulation of cell migration” was identified in clusters upregulated at 0.5% O_2_ and below (Fig. 3A) [48]. Additionally, genes ascribed to “negative regulation of cell adhesion” were significantly downregulated across LTHY, consistent with the increase in cell aggregation we observed at 0.5% O_2_ and below. Finally, these findings were further corroborated through Gene Set Enrichment Analysis (GSEA) analyses. While GSEA analyses revealed a significant enrichment for hypoxia adaptation (Fig. 3B), as expected, there was greater enrichment for EMT hallmark genes (Fig. 3C) as measured by Normalized Enrichment Score (NES), strengthening our hypothesis that LTHY induced EMT.

Contrasting with these findings were some GO term analyses for clusters upregulated at 0.5% and 0.1% O_2_. Indeed, genes associated with negative regulation of TGFβ signaling, SMAD phosphorylation, and BMP signaling, all pointing to an inhibition of EMT, were enriched (Fig. 3A, D). This suggests a dampening of TGFβ signaling, a major EMT-inducing pathway, at the late LTHY stages [49, 50]. In line with this was the upregulation of negative TGFβ signaling genes (Fig. 3D). Together, these data suggest a non-canonical, TGFβ-independent induction of the EMT signature during LTHY [51].

To determine if this was the case, we first examined canonical EMT drivers. Most of the classical EMT-driving genes were either not expressed at all, or not differentially expressed across LTHY, suggesting they were not driving the LTHY-induced EMT-like state of the cells (Fig. S3A). However, significant upregulation of hallmark EMT effector genes (s100a4, Fn1, Col4a1, Col4a2, Col4a1, and others) was observed, confirming the EMT-like state of the cells (Fig. 3E). In addition, Vim was also upregulated at both 0.5% O_2_ and 0.1% O_2_ (Fig. S3B), although this upregulation was not significant (lowest padj = 0.13) and doesn’t match the increase observed at the protein level (Fig. 1F). This discrepancy, however, may be explained by the reduction in miR146a-5p, a known repressor of vimentin [52, 53]. It may also be that like miR-210-3p, Vim is also moderately upregulated at 5% O_2_ relative to normoxic conditions, thereby making the increases in expression non-significant. Finally, the E-to-N Cadherin switch, another EMT hallmark, was also dysregulated at the mRNA level; as E-cadherin (Cdh1) was not sufficiently expressed, and N-cadherin (Cdh2) was only moderately upregulated across LTHY, contrasting with results obtained at the protein level (Fig. S3C, Fig. 1F). Despite this, our data and analyses confirm the EMT-like state of the cells induced during LTHY and highlight potential non-canonical EMT pathways.

We therefore investigated potential drivers for this non-canonical EMT induction. To do so, we filtered for transcription factors (TFs) expressed at 0.5% O_2_ and below and cross-referenced them to EMT, allowing us to identify candidate drivers (Fig. 3F). Some of the TFs identified were of interest as they are related to EMT. However, most did not correlate with downstream effectors known to impact EMT/MET or follow the onset on EMT-like morphological changes. Indeed, while ETV4/5 have links to glycolytic adaptation and EMT, they have been shown to act through CXCR4 and S100A8/9 axes, both of which are not expressed in our system (zero mapped reads). CEBPD, another known TF with ties to EMT, has been shown to regulate Cdh1/2 as well as ZO-1 (Tjp1) expression in relation to TGFβ-driven EMT (Fig. S3C). However, these target genes did not change in expression in our setting pointing away from a canonical role for CEBPD in LTHY-induced EMT [54]. Furthermore, both Klf2 and Atoh8, which are considered as EMT inhibitors, were upregulated during LTHY adaptation conflicting with our morphological and biochemical assessment of B16 adapting to LTHY [55, 56]. However, Klf2 has been mostly characterized in TGFβ-driven EMT as a TGFβ signaling inhibitor, which we have here, and yet LTHY-adapted cells display a clear EMT transcriptional phenotype. Klf2 has also been shown to promote quiescence, which we did not observe throughout LTHY [56]. Finally, Atoh8 is another known EMT inhibitor known to act through SMAD3 to induce senescence. However, due to evident negative regulation of TGFβ signaling and continual cell growth throughout LTHY, it too was cast aside [57–59]. In contrast, Wt1 is well established in the literature as an EMT driver in both developmental and cancer settings and was the most significant DEG (>24 fold-change) in the dataset (Fig. 3F). In addition, Wt1 expression has been shown to be regulated by hypoxia through HIF1α [60]. However, its role in being involved in hypoxia induced EMT had yet to be characterized.

### Gradual adaptation to severe hypoxia induces a novel Wt1 transcript

**Fig. 4:**
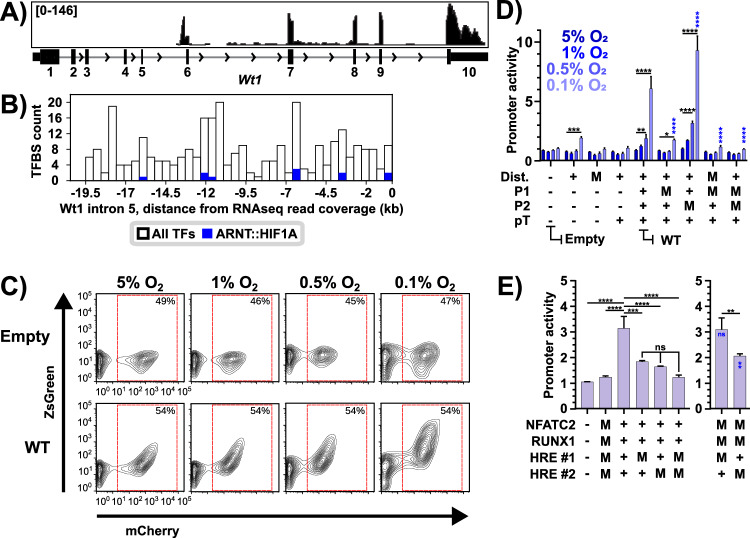
The long-term hypoxia adaptation induces novel truncated Wt1 mRNA transcripts from an intronic HRE-driven promoter. **A** LTHY read coverage of the *Wt1* locus, at 0.1% O_2_ of the LTHY time course, generated in IGV. Number ranges are coverage depths at the nucleotide level. Histogram is representative of replicates (*n* = 2, n2 shown). Introns 1–4 are condensed for visual clarity. **B** Transcription Factor Binding Site analysis of murine *Wt1* intron 5 from beginning of intron 5 to beginning of RNAseq read coverage for Wt1. Only considered TFBSs with a score ≥ 0.95. Intron 5 sequence is broken into 40 bins, ~500 bp/bin. Analysis done using TFBStools in R. **C** FACS samples of the empty promoter-reporter construct, and the wild-type (WT) across the LTHY time course. Gate represents mCherry+ gate used for promoter activity calculations. FACS plots are representative of their triplicates. **D** Functional investigation into tWT1 promoter subregions. “Dist”: Distal region. “P1”: Proximal subregion 1. “P2”: Proximal subregion 2. “pT”: Poly-Thymine stretch. +: DNA region is present. −: DNA region is not present. M: DNA region has specific TFBSs scrambled. Promoter activity calculated using a ratio ZsGreen expression in transduced cells relative to untransduced cells, normalized to their normoxic counterparts. Significance calculated using 2-way ANOVA with Tukey’s multiple comparisons. Black stars represent intra-construct statistical comparisons; only reporting statistics relative to 5% O_2_. Blue stars represent significance relative to WT at 0.1% O_2_. Other comparisons are not shown for visual clarity. **E** Functional investigation into the P1 subregion of the tWt1 promoter at 0.1% O_2_. Promoter activity was calculated as in **D**. Statistics are a 2-way ANOVA with Tukey’s multiple comparisons test. Black stars represent statistical comparisons. Blue stars represent significance relative to WT at 0.1% O_2_. Other comparisons not shown for visual clarity. **D**, **E** “-“: an absence of subregion. “+“: presence of wild-type sequence. “M”: Transcription factor binding sites listed in Fig. S4F are scrambled. *p < 0.05. **p < 0.01. ***p < 0.001 ****p < 0.0001.

Given the diversity of WT1 mRNA isoforms in humans and mice, we examined the RNAseq read coverage for the *WT1* locus to identify which isoforms were expressed [14] (Fig. 4A, Fig. S4H, I). Surprisingly, there was no read coverage for the first five exons of *WT1*, with all reads mapping to the exon 6-10 region of the gene (Fig. S4A). We confirmed that unexpressed exons were present and without mutation in the B16-HG genome, confirming the genomic integrity of the locus (Fig. S4B and supporting material). RNAseq read coverage began 195 bp upstream of exon 6, within intron 5, suggesting that transcription was being initiated from a previously undescribed transcription start site (TSS). To investigate a potential promoter region upstream of the RNAseq read coverage, we performed a Transcription Factor Binding Site (TFBS) analysis across the entire 20 kb intron 5 sequence, considering only the transcription factors expressed at 0.5% O_2_ (Fig. 4B). With this approach, we identified several HIF1 binding sites (HREs) within intron 5 and determined that all these HREs were accessible to Hif1 by ChIP-qPCR (Fig. 4B, Fig. S4C). In addition, several other TFBSs for transcription factors expressed in the B16-HG cell line at 0.5% O_2_ were identified throughout the intron, suggesting extensive transcriptional regulation within intron 5 (Fig. S4D).

Given the hypoxia-dependent nature of Wt1 upregulation and the binding of Hif1 to intron 5 HREs, we investigated whether the genomic region upstream of the RNAseq coverage constituted a functional promoter. To do so, we developed a reporter construct, which constitutively expresses mCherry and where ZsGreen expression is driven by the putative promoter or variants thereof (Fig. S4E). The putative promoter encompassing 551 bp upstream of the TSS, was broken down into four distinct regions (Fig. S4F). Upstream from the TSS, the first region is the poly-thymine (PolyT) stretch due to its sequence composition. Beyond this is the proximal region, which was subdivided into P1 and P2, and the distal region, which contains a long poly-AG stretch. It is important to note that we observed no changes in the expression of all the transcription factors associated with the TFBSs in these regions, apart from Nr4a2 downregulation (Fig. S4G).

To gain insights into the transcriptional regulatory ability of each subregion and TFBS, we built a panel of promoters consisting of either subregion deletions or TFBS mutation. We then performed a transcriptional activity screen in B16 cells and monitored mCherry and ZsGreen expression across LTHY. As control, the cells were cultured in parallel in normoxic conditions. As expected, the “empty” version of the promoter-reporter system did not respond to LTHY (Fig. 4C, D). Contrastingly, the “wild-type” putative promoter induced ZsGreen in a pattern that mimicked the kinetics of Wt1 during LTHY, demonstrating its role as a hypoxia-sensitive promoter (Fig. 4C, D). Conversely, when all the P1 TFBSs were mutated, we observed a significant and substantial reduction in ZsGreen levels, suggesting its role as the main driver of LTHY-induced Wt1 expression. Intriguingly, when the P2 TFBSs were mutated, expression levels of ZsGreen significantly increased, suggesting a role as a negative regulator of transcription (Fig. 4D). The distal region also appears to possess some transcriptional activity, as there was a small but significant increase in ZsGreen levels when it was the only constituent of the putative promoter.

Finally, we mutated each HRE within P1 to assess their individual role in regulating tWt1 expression during LTHY. Our data indicates that while both HREs contribute to the promoter activity, HRE #2 seems to possess greater transcriptional activity as a standalone element (Fig. 4E). Mutation of the RUNX1 and NFATC2 sites minimally altered ZsGreen expression in the context of HRE-deficient conditions indicating they were non-functional in this context. Together, our data establishes the genomic region within intron 5 of murine *WT1* as a bona fide hypoxia-sensitive promoter through necessary and sufficient HIF1 binding sites, can initiate transcription of Wt1 at 0.5% O_2_, and increase transcriptional activity as cells adapt to more severe hypoxia.

### Identification and characterization of truncated Wt1 transcripts

**Fig. 5:**
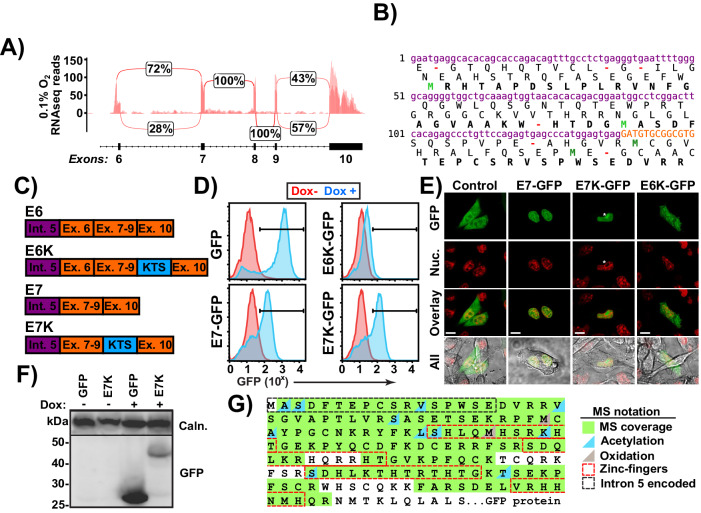
Novel truncated Wt1 transcripts encode efficiently translated proteins that accumulate in the nucleus. **A** Splicing events observed in LTHY data. Percentages are the average between replicates, coverage depths are overlaid. **B** Potential open reading frames (ORFs) derived from the tWt1 intron 5 sequence in E7 isoforms. Purple: Intron 5 derived sequence. Orange: Exon 7 derived sequence. Bold: Canonical WT1 ORF (third ORF). Bright-green/dark-green: In/out of frame start codons. Red: Stop codons. **C** Possible tWt1 isoforms. Purple: Intronic sequence. Orange: Exonic sequence. Blue: KTS motif. **D** GFP levels of DOX-induced expression of tWT1-GFP isoforms in B16 cells. **E** Microscopy images of tWT1-GFP fusion constructs. Nuclear staining was performed using Hoechst 33342 (Thermo FIsher: H1399) as per the manufacturers protocol. **F** Western Blot of HEK cells expressing DOX inducible GFP or E7K-tWT1-GFP. Top: anti-Calnexin. Bottom: anti-GFP. **G** Mass Spectrometry (MS) coverage of E7K-tWt1 purified from HEK cells. Refer to the legend for full annotation.

Next, we investigated the functionality of the novel truncated Wt1 (tWt1) transcripts as RNAseq coverage analyses revealed the presence of exonic spikes and read junctions suggesting a mature mRNA. These analyses also revealed a novel splicing event joining the 3’ end of intron 5 to the 5’ end of exon 7 leading to a novel RNA which excludes exon 6 (Fig. 5A). Canonical exon 6 to exon 7 splicing was also observed in some transcripts but constituted the minority of splicing events. Our analyses also identified the known KTS splicing event, which introduces a lysine-threonine-serine motif between zinc fingers 3 and 4 of WT1 between exons 9-10, at a near 1:1 frequency, in line with previous reports [14, 61]. The novel splicing site within intron 5 occurred 58nt upstream of exon 6 (Fig. S5A). Interestingly, when either splicing event occurs, it adds an intronic sequence to the beginning of the tWt1 mRNA transcripts upstream of exon 6 or exon 7, and introduces potential start codons (Fig. 5B, Fig. S5A). Based on the observed splicing events, there are four possible mRNA species, named for their first canonical exon (E6, E7), and the presence of the KTS motif (E6K, E7K) (Fig. 5C).

To determine whether any of these tWt1 transcripts could be translated to produce functional protein, we fused each of them to a C-terminal, ATG-deficient, GFP in doxycycline-inducible lentiviral vectors (Fig. S5B). This ensures fluorescence only occurs via an in-frame functional start codon within the tWt1 transcript (Fig. S5C). Cell lines stably expressing the various tWt1 transcripts were treated with doxycycline and analyzed by FACS to determine the level of tWt1-GFP expression, while subcellular localization was determined by confocal microscopy. (Fig. 5D, E). Following Dox induction, both the E7 and E7K variants produced a robust GFP signal and were localized to the nucleus, as expected based on WT1, with E7K displaying clear nucleolar accumulation, a known attribute of KTS + WT1 isoforms [62]. In contrast, E6K-GFP failed to generate substantial GFP expression or nuclear localization, suggesting non-functionality for both E6 isoforms.

Western blot analysis of the E7K -GFP fusion protein, which showed a substantial band at 48kDA, suggested translation initiation within the intron 5 derived sequence, which was confirmed by mass spectrometry (MS) analyses of immunoprecipitated E7K-GFP (Fig. 5F, G, Fig. S5E). Interestingly, translation initiation of the E7 polypeptide correlated with the Kozak context of the in-frame start codons, with the strongest Kozak signal at the second intron 5 derived in-frame start codon (Fig. S5D). Kozak strength of the in-frame ATGs also explains lack of E6-GFP translation, as the putative intron 5 derived ATG is out of frame with tWt1 in the E6 variant, and no other strong in-frame ATGs are present in E6 (Fig. S5D).

### LTHY-induced tWt1 retains DNA-binding and links to EMT

**Fig. 6:**
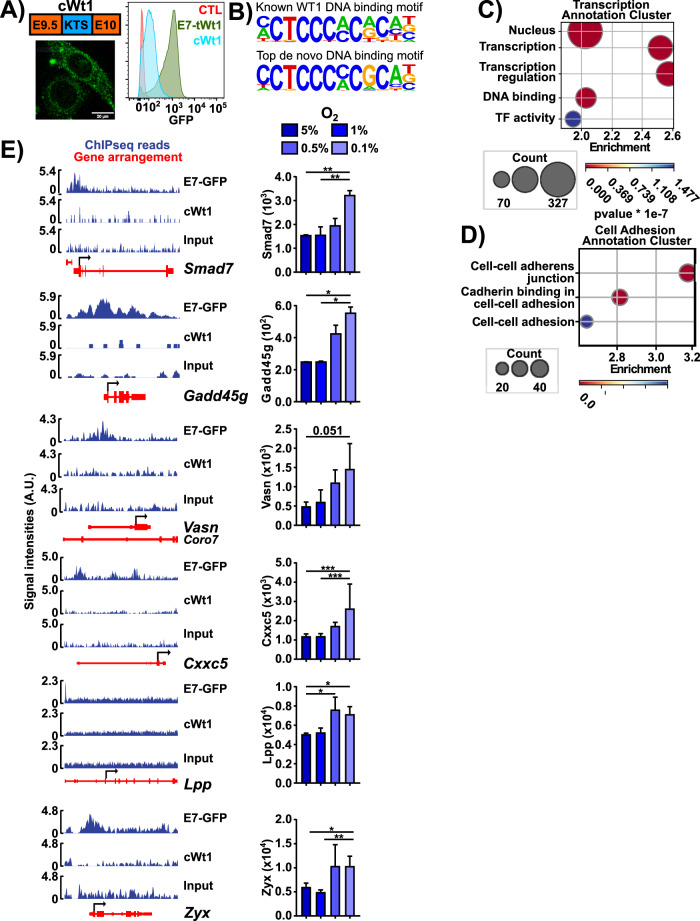
Novel E7-tWT1 isoform retains DNA binding ability and is associated with genes involved in EMT. **A** Left: top: schematic of cWt1 CDS. GFP was linked C-terminally as per the E7-GFP construct. Bottom: microscopy image of cWt1 under Dox induction. Right: GFP induction levels of cWt1 relative to E7-GFP. Induction was performed after 36 h of incubation at 0.5% O_2_, as per the LTHY protocol. All Dox inductions were performed at 2 µg/mL. **B** Known and de novo TF motif analysis of E7-K tWT1 ChIPseq data. The known motif *p*-value = 1e−78, is found in 36% of called peaks. The de novo motif *p*-value = 1e−105, motif is found in 30% of ChIPseq peaks. **C**, **D** Functional annotation bubbleplots of ChIPseq called peaks. **E** E7-tWT1 ChIPseq coverage and LTHY RNAseq expression profiles for genes of interest. cWt1 and input chromatin were used as negative controls. Black arrow denotes CDS start. * denotes relative significance as calculated by DESeq2 Benjamini-Hochberg adjusted *p*-value (padj). *padj < 0.05, **padj < 0.01, *** padj < 0.001.

Due to the unambiguous nuclear localization of E7-GFP, we sought to validate its functionality. To do so, we performed ChIPSeq with anti-GFP on E7- eGFP B16 cells after 36 h at 0.5% O_2_ as per the LTHY protocol. As controls, we used both input ChIP DNA, and a critically truncated version of Wt1 (cWt1) which loses nuclear localization and therefore does not bind to DNA (Fig. 6A). Our ChIPseq analyses identified 865 genes (Table 1). Motif analysis showed significant enrichment for the known Wt1 motif, which was found in 36% of peaks, and a de novo motif in 30% of peaks, which only differed in some preferred nucleotides (Fig. 6B). Regardless of whether or not they contained the WT1 binding motifs, peaks were predominantly found near the TSS, suggesting that E7-tWt1 acts as a promoter, similar to WT1 (Fig. S6A) [63]. Functional annotation analyses revealed significant enrichment for transcription and cell adhesion annotation clusters, with a specific enrichment of cell-cell adhesion annotations (Fig. 6C, D, Fig. S6B).

**Table 1:** Breakdown of ChIPSeq genomic locations.

Region type	All ChIPseq peaks	DAVID filtered peaks	Peaks with Wt1 motif
promoter-TSS	472	428	181
non-coding	17	7	4
Intergenic	146	118	37
intron	305	278	106
exon	62	58	21
5’ UTR	89	88	28
3’ UTR	7	6	1
TTS	11	10	3
Total peaks	1109	993	381
Total genes	893	865	369

ChIPSeq peak calls performed using MACS. Annotated peak table was used to collect gene symbols. Gene symbols were passed to DAVID for GO term enrichment. Wt1 binding motif was determined using an in-house analysis pipeline.

We also identified several genes associated with EMT, which had expression kinetics matching those of E7-Wt1 and the appearance of EMT-like features (Fig. 6E). Indeed, *Zyx*, *Lpp*, and *Vasn* are known cellular motility genes, and *Gadd45g*, *Cxxc5*, and *Smad7* can influence EMT through transcriptional regulation. *Cxxc5* is a known WT1 (-KTS) target gene, providing additional strength to the validity of the dataset, functionality of E7-tWt1 and its potential role in mediating LTHY-induced EMT [64].

### Identification of tWT1 in human cancers and prognostic value

**Fig. 7:**
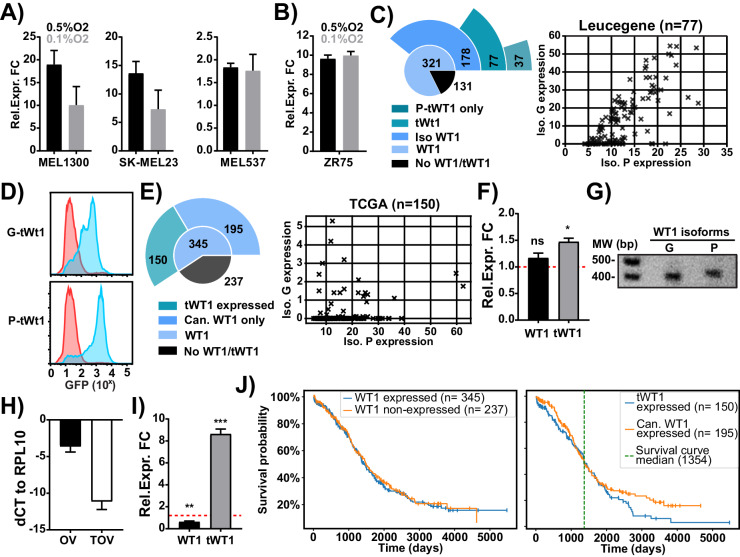
Novel tWT1 transcripts are expressed in human cancers and are indicative of poor long-term survival in ovarian cancers. **A** tWT1 expression in human melanoma cell lines undergoing LTHY as measured by qPCR. Expression presented as relative to fold change (FC) to the 5% O_2_ condition **B** tWT1 expression in ZR75 cells during LTHY incubation in the absence of estradiol (E2), relative to the 5% O_2_ timepoint. **C** Left: sunburst plot of Leucegene samples based on WT1 gene and isoform expression. Iso WT1 denotes the number of samples where WT1 isoform calling could be performed. Right: Breakdown of tWT1-G/P expression levels in tWT1 expressing Leucegene samples. Isoform calling was done using km and the isoform specific difference between G and P, using km’s Expectation-Maximization algorithm. **D** tWT1-GFP expression levels as determined by FACS in HEK293T cells. tWT1-GFP expression was induced by 2 µg/mL DOX for 36 hs at the 0.5% O_2_ timepoint of the LTHY protocol. **E** Left: Sunburst plot of TCGA-OV samples by WT1 isoform expression. Right: Breakdown of tWT1-G/P expression levels in tWT1-G/P expressing TCGA-OV samples. Isoform calling was done using km and the isoform-specific difference between G and P, using km’s Expectation-Maximization algorithm. **F** WT1 and tWT1 mRNA expression in human ovarian cancer cell line OVCAR3 after completing the LTHY adaptation, relative to normoxia. **G** RT-PCR of human tWT1 isoforms from normoxic OVCAR3 cDNA. **H** Delta-CT values calculated from OVCAR3 and TOV3291G cells in normoxic conditions using RPL10 as housekeeping gene to assess baseline expression. **I** WT1 and tWT1 mRNA expression in human ovarian cancer cell line TOV3291G after completing LTHY, relative to normoxia. Data presented as relative expression to normoxic condition, **J** Survival curve analyses for TCGA-OV (ovarian cancer) based on WT1 expression subsets. Left: Kaplan-Meier estimation survival curve of TCGA-OV samples, comparing samples which express any isoform of WT1 versus those with no WT1 expression. Curves are not significantly different (p = 0.26). Right: Kaplan-Meier estimation survival curve of TCGA-OV samples, comparing samples which express tWT1 isoforms (isoforms G or P) versus those which exclusively express canonical WT1 isoforms. Two-sided *p*-value of the whole curve is 0.12. Two-sided *p*-value of post-median data (126 observations) is 0.039. WT1 expression and isoform calling were determined by detection of exons 1, 1a, 2, 4, 7, and isoform G exon 1 by km.

Finally, we moved towards determining whether induction of tWt1 in cancer cells undergoing long-term and severe hypoxia adaptation could be observed in human cancers and whether we could infer a prognostic value to its expression considering its link to EMT. To do so, we performed qPCR analyses on human melanoma and breast cancer cell lines undergoing LTHY adaptation using primer pairs that enable us to determine the expression of canonical WT1 or tWT1. Our results indicate that most tumor cells tested significantly induced the expression of tWT1 following LTHY adaptation, except for the MEL537 melanoma cell line which constitutively expressed tWT1 (Fig. 7A, B, Fig. S7A, B). This points to a generalized mechanism of expression regulation across species and cancer types. Interestinlgy, the breast cancer cell line tested displayed significant increase in tWT1 induction during LTHY in the presence of the ERα agonist estradiol (E2) (Fig. S7B).

Next, we investigated whether LTHY-induction of tWT1 mRNA transcripts in humans was similar to that observed in mice. The genomic landscape of the *WT1* locus is similar between humans and mice, suggesting potential similarities in intragenic regulation of transcription (Fig. S7C, top). We first analyzed RNAseq data from AML-patient data from the Leucegene database due to its known prevalence of WT1 expression and high depth of sequencing, which facilitates the identification of splice variants using an alignment-free Kmer approach [65, 66]. This enabled us to identify a previously characterized WT1 isoform, annotated as G (G-tWT1), and a new isoform we termed P (P-tWT1) (Fig. S7C). Both isoforms arise from an intron 5 TSS, where G-tWT1 has a splicing event within intron 5 and exon 6, while P-tWT1 displays a continuous sequence from intron 5 into exon 6 (Fig. S7C) [67]. A breakdown of the Leucegene dataset revealed that most patient samples express WT1 at the RNA level, with 77 samples exclusively expressing either tWT1 isoforms. Of those 77 samples, 37 exclusively expressed P-tWT1. (Fig. 7C, left). When plotted against each other, we observed a bias towards the expression of P-tWT1 over G-tWT1 (Fig. 7C, right). While the added intronic sequence in G-tWT1 does not introduce an in-frame ATG like E7-tWt1, Dechsukhum and colleagues previously reported that translation initiated through a non-canonical CUG start codon found in the added intronic sequence (Fig. S7D) [67]. In contrast, the inclusion of the elongated intronic sequence in P-tWT1 introduces an in-frame ATG with similar Kozak strength to the functional ATG in the murine E7-tWt1, suggesting that it could be translated in a similar fashion (Fig. S7D, E). Combined with the expression bias in tumor samples, P-tWT1 appears to be the more relevant isoform. We validated this by fusing the G- and P-tWT1 RNA sequence to ATG-deficient GFP under the control of a Dox-inducible promoter as done previously for murine tWt1. The new constructs (G-tWT1 and P-tWT1) were transduced into HEK293T cells, and we monitored GFP expression by flow cytometry and characterized the protein expression profiles by Western blot (Fig. 7D, Fig. S7F). Not only was P-tWT1 more highly expressed than G-tWT1, but translation resulted in a fusion protein similar in size, as expected from the RNAseq, as that of the functionally active murine E7-tWt1 and detected with both the GFP and a C-terminally conserved epitope within exon 7 (Fig. S7F).

We next investigated the expression of tWT1 isoforms across cancer patient samples and determined their value as prognostic markers through The Cancer Genome Atlas (TCGA) database. Surprisingly, within TCGA, tWT1 isoforms were exclusively identified in ovarian cancer (TCGA-OV), which is also the subset with the highest WT1 expression [68]. In contrast to the Leucegene dataset, no samples could be identified with exclusive tWT1 expression, as canonical WT1 expression was always concomitant (Fig. 7E, left). Additionally, the tWT1 isoform expression bias towards P-tWT1 was much more pronounced in ovarian cancer when compared to Leucegene (Fig. 7E, right). As ovarian cancers are known for being highly hypoxic, we tested whether ovarian cancer cell lines adapting to LTHY would also display increases in tWT1 expression and undergo EMT. For this, we first tested the well-characterized cancer line OVCAR3. There was no significant change in WT1 and tWT1 mRNA expression, most likely due to its high expression in normoxic conditions (Fig. 7F, H). Indeed, we could readily amplify both the G- and P-tWT1 isoforms from OVCAR3 cells in normoxia (Fig. 7G). We also tested another ovarian cancer cell line isolated from a primary tumor, and previously characterized by Sauriol et al. for having WT1 expression as determined by immuno-histochemistry staining from biopsies and Western Blot analyses of fresh isolates [69–71]. Contrary to the OVCAR3 cell lines, the TOV3291 cells had very little expression of tWT1 in normoxic conditions, as assessed by qPCR, but we observed a substantial and significant upregulation following LTHY adaptation (Fig. 7H, I). Despite differences in tWT1 induction between the two cell lines, they both engaged in an EMT-promoting transcriptional program following LTHY as determined by qPCR, with the primary tumor-derived TOV3291G cells displaying a more striking signature. (Fig. S7G).

Finally, we determined the prognostic values of WT1 and tWT1 for ovarian cancer patients using TCGA-OV. While overall survival probabilities couldn’t be predicted through WT1 expression, patients also expressing P-tWT1 seemed to display worse long-term survival probabilities (Fig. 7J). Dissection between overall and long-term survival probabilities was made possible by calculating survival significance using a sliding start date window, which shows a large window of significance past the minimal median survival date (1354 days). Using this approach, we were able to determine to determine that P-tWT1 expression is a significant negative prognostic marker in ovarian cancer for long-term survival (*p* < 0.05), but not overall survival (*p* = 0.12) (Fig. S7H).

Together, our data demonstrate the existence of a novel WT1 isoform (P-tWT1) in humans, which closely resembles the murine E6-tWt1 in mRNA structure but possesses a productive in-frame start codon within the additional intronic sequence similar to E7-tWt1, and that expression of this WT1 isoform correlates with a negative long-term outcome for ovarian cancer patients.

## Discussion

There is a need to better understand tumor cell adaptation to sustained and severe hypoxia to grasp its impact on tumor cells and patient outcome. Here, we provide a new cell culture method, LTHY, developed to mimic the gradual onset of severe hypoxia, and recapitulate the conditions observed in vivo. Despite recent advancements in hypoxic incubation protocols, our method combines both duration and severity to mimic tumor onset and progression [9]. LTHY spontaneously engages EMT-like changes, which can be observed both morphologically and transcriptionally. However, these changes do not occur through pathways implicating known EMT external drivers such as TGFβ, signaling suppression, nor canonical EMT-associated transcription factors. Yet, expression of many EMT effector genes and miRNAs corroborates the initiation of EMT and agrees with previous work demonstrating that hypoxic adaptation, at 0.5% and below, induces an increase in cell motility in vivo suggestive of EMT [72].

Indeed, the EMT-like morphological changes observed at late stage LTHY were corroborated by with phenotypic changes such as the expression profiles of Vim, E-Cad and N-Cad and a clear EMT-promoting miR signature solidifying our assertion of spontaneous EMT [20–25]. In addition, we identified several other miRNAs with expression changes at the later stages of LTHY, but with unknown pathways linking them to our hypoxia-induced EMT-like signature. Such miRNAs include known suppressors of EMT, such as miR34b/c, shown to suppress EMT-like features in lung adenocarcinoma under normoxia, or TGFβ-dependent EMT regulators, such as miR-199a-5p [24, 73]. Furthermore, our analyses revealed the B16 cells did not differentially express the miR-200 family of miRNAs, which are known modulators of EMT [26]. These discrepancies may be due to the type of EMT induced during these assays, which may differ greatly from ours, and may reflect the different routes that cells take to induce EMT [74]. A combined analysis of miRNA expression, expected targeting, and mRNA expression is needed to both properly identify functional miRNAs to further elucidate their mode of action in LTHY-induced EMT.

Our work has also enabled the identification of a novel Wt1 isoform transcribed from a previously undescribed promoter region within intron 5 in both mouse and human loci, pointing to a conserved mechanism of induction. We show that the intronic promoter activity is HIF1-dependant in mice, with additional regulation provided by other factors. Additionally, this region coincides with a candidate cis-regulatory elements in both mice (EM10E0704920) and humans (EH38E1530575), further validating its functionality [75]. This finding identifies the second hypoxia-dependent *WT1* promoter, and the first arising from an intronic region [60]. Intriguingly, induction of tWt1 expression occurred in the absence of additional increases in HIF1α stabilization, as assessed with our HIF1α-eGFP reporter line, suggesting additional rewiring of the transcriptional program beyond initial HIF1 activity. However, it is important to note that the level of HIF1 stabilization in the later stages of LTHY may be underestimated in our assay, as eGFP requires oxygenation to possess fluorescence activity [76, 77]. Nonetheless, the tWt1 intronic promoter was only active at 0.5% O_2_ and below, despite HIF1 being active at earlier time points. In fact, we see dramatic transcriptomic changes across oxygen conditions in our RNAseq datasets, despite stability in overall HIF1 levels, strongly suggesting additional layers of transcriptomic regulation in response to LTHY. This may provide an explanation as to how the expression of some HIF1 targets, like miR210, do not continue to increase as hypoxia becomes more severe. This may also be the result of epigenetic changes across LTHY, as a clear signature of effectors was identified in RNAseq, which may impact HIF1 activity, but would have to be further studied to be validated. However, this may not be the case for the tWt1 promoter, as our assay removes it from the local epigenetic context, yet it retained the transcriptional kinetics of tWt1 during LTHY. Finally, it may be that specific Hif1α/β post-translational modifications (PTMs) are driving different transcriptional preferences, as previously described [78].

Changes in HIF1 transcriptional activity are, however, not likely due to the effect of the dominant negative FIH (HIF3α), as Hif3α is not significantly expressed across LTHY. Less than 100 reads mapped to the Hif3a locus in any sample, with none spanning exon-exon junctions (data not shown). Alternatively, it may be that differences in transcriptional regulation are the result of a reduction in negative regulator activity, allowing for the de-repression of various genes, as was shown within the tWt1 promoter P2 sub-region. Nevertheless, HIF1 activity produces the dominant E7-tWt1 isoforms, where the novel splicing event introduces an intron 5-derived ATG with a strong Kozak context into the frame with the remaining WT1 CDS, and results in translated truncated Wt1 protein isoforms. Counterintuitively, this functional ATG is the second in the transcript, with the first ATG generating a small upstream ORF. Interestingly, upstream ORFs are a known mechanism for repressing normoxic translation of downstream ORFs while enhancing their translation under hypoxia. This may explain the hypoxia-dependent increase of E7-tWt1 expression, as this mechanism is known to also occur in the case of human EPO [79]. Finally, E7-tWT1 PTMs were identified using mass spectrometry, extended beyond those described in the literature, which could also confer hypoxic stabilization [80].

Our results also demonstrate that E7-tWt1, although heavily truncated, retains much of its function and regulates the expression of genes linked to gene transcription and cell-cell adhesion, two functional signatures also obtained by Ullmark and colleagues, using WT1 KTS(-) as bait in ChIPseq experiments [63]. However, investigating protein binding partners may shed additional light on E7-tWt1 functionality, as the lack of canonical N-terminus would alter the pool of interactors [81]. Additionally, our ChIPseq data suggests that tWt1 may be involved in hypoxia-induced EMT, as several genes linked to EMT were identified as targets, and WT1 is a known mediator of EMT. Finally, further investigation into E7K-tWt1 RNA binding is warranted, given the known RNA binding ability of KTS + WT1 isoforms and their implication in cancer progression [82].

We also provide compelling evidence of tWT1 isoform expression in human cancers as all human cancer cell lines tested either expressed them constitutively (MEL537, OVCAR3) or induced their expression during LTHY (MEL1300, SK-MEL23, ZR75, TOV3291G) suggesting a conserved mechanism of action. Further identification of a novel P-tWT1 isoform in AML and ovarian cancer adds to a long list of previously identified human WT1 isoforms, but only the second of its kind that stem from an intragenic TSS, as most isoforms arise from alternative splicing combinations [83]. Although P-tWT1 resembles murine E6-tWt1 in sequence arrangement with a continuous sequence from TSS into exon 6, it contains a potent in-frame ATG like E7-tWt1, which resulted in functional protein translation. This suggests a convergent evolution in cancer, where cancer cells from different species attempt to express a functional truncated version of WT1 through different mechanisms [84].

Finally, within TCGA-OV, tWT1 expression was found to be a negative prognostic marker for ovarian cancer for late-term survival using a new, non-biased approach enabling differential analysis of early and late survival probabilities. Curiously, TCGA-OV was the only TCGA dataset containing tWT1 expression, and correlated with the higher level of WT1 expression in this cancer type compared to others, where it was shown to promote EMT under hypoxic conditions [85, 86]. Identification of tWT1 only in TCGA-OV may be due to the prevalence of hypoxia in this cancer, as it is often diagnosed late into progression and therefore would have a higher degree of tumor hypoxia, thereby increasing the chances of obtaining biopsies derived from hypoxic microenvironments [87]. This is particularly important in the case of TCGA due to the sequencing depth per patient sample. In order to identify alternative transcripts, a higher sequencing depth is required than compared to gene-level expression analyses. Therefore, the TCGA database may be limiting both in terms of tumor microenvironmental representation, as well as transcriptomic representation. Deep RNAseq analyses enabling the discovery of alternate transcripts or mutations require a higher number of reads (typically more than 100 million reads per sample) [88]. While sufficient for gene-level expression analyses, the depth provided by TCGA samples may be insufficient for accurate tWT1 isoform calling [85]. As an example, we readily identified P-tWT1 in AML patient samples from the Leucegene dataset, which contains an average of 200 million read per sample, but not in AML samples from TCGA [65]. In conclusion, while further work is needed to elucidate the molecular tWT1 isoforms and their functions, its potential as a novel therapeutic target may be of particular interest for immunotherapy as the peptide obtained through translation of the added intronic sequence could provide a cancer-specific cryptic antigen [89].

### Supplementary information

Supplemental material and methods

Supplemental figures

## Data Availability

All RNAseq, MS, and ChIPseq data can be accessed at: 10.5061/dryad.cz8w9gjb4.

## Code availability

Methods for in-house analyses are described in Materials & Methods. Code may be shared upon request.
