# Supplementary material for: Long-term severe hypoxia adaptation induces non-canonical EMT and a novel Wilms Tumor 1 (WT1) isoform
Source: Cancer Gene Ther. 2024 Jul 8;31(8):1237–50. doi: 10.1038/s41417-024-00795-3 (PMC11327107; doi:10.1038/s41417-024-00795-3)
Supplement: Supplementary file 1 — Supplemental material and methods [file 41417_2024_795_MOESM1_ESM.pdf]

<u>Supplemental Materials and Methods:</u>

<u>Additional cell lines and related methods</u>

tWt1 promoter expressing B16 cells: The WT versions of the distal and proximal regions of the tWt1 promoter were amplified through genomic PCR, as was the mutated version of the Distal subregion. The various P1/P2 mutated subregions were generated as gBlocks by IDT (Supplemental table 1).

Supplemental table 1: Murine tWt1 promoter reporter P1 & P2 sequences.

P1	P2	gBlock sequence (uppercase: mutated sequences)
MUT	MUT	gagagggagagagagacagagttaacttgatcATAGTCTctgatcagtggat<br>gagagGCCTGGACCTACACGacccccactcccttctgaTAGGAGCGcatctc<br>ttgtaaAGTATCCAactttcttggtgctatctggtaagaactgagtcatgag<br>gtgccgacaaaaccccagtgtacttccctcgaagcctgcagcattctgctca<br>ttcctgagatgctcctgctggggccactgctcagatgctTTTCGGAGTTAtg<br>cctgggatgagatcctgacactCGAAGGAGACGCGTgcattagatcgta
WT	MUT	gagagggagagagagacagagttaacttgatctggaaaactgatcagtggat<br>gagaggagccacagacgtgcacccccactcccttctgaggacgtgacatctc<br>ttgtaaatgtcatcactttcttggtgctatctggtaagaactgagtcatgag<br>gtgccgacaaaaccccagtgtacttccctcgaagcctgcagcattctgctca<br>ttcctgagatgctcctgctggggccactgctcagatgctTTTCGGAGTTAtg<br>cctgggatgagatcctgacactCGAAGGAGACGCGTgcattagatcgta
MUT	WT	gagagggagagagagacagagttaacttgatcATAGTCTctgatcagtggat<br>gagagGCCTGGACCTACACGacccccactcccttctgaTAGGAGCGcatctc<br>ttgtaaAGTATCCAactttcttggtgctatctggtaagaactgagtcatgag<br>gtgccgacaaaaccccagtgtacttccctcgaagcctgcagcattctgctca<br>ttcctgagatgctcctgctggggccactgctcagatgctggtaaacaacttg<br>cctgggatgagatcctgacactgtggccttACGCGTgcattagatcgta
WT	WT	gagagggagagagagacagagttaacttgatctggaaaactgatcagtggat<br>gagaggagccacagacgtgcacccccactcccttctgaggacgtgacatctc<br>ttgtaaatgtcatcactttcttggtgctatctggtaagaactgagtcatgag<br>gtgccgacaaaaccccagtgtacttccctcgaagcctgcagcattctgctca<br>ttcctgagatgctcctgctggggccactgctcagatgctggtaaacaacttg<br>cctgggatgagatcctgacactgtggcctt
Dist WT		atccaggtgtgtttgtaatcccagcactgaagaaatggagacaatggagaca<br>gggggaatctctagaattgctgggcagccagcctagctgaattggtgagatc<br>taggctcagggagagactcagttacaaaagatagaaggaagagaaaccaagg<br>aaggtactggatgtctagttagggatgcacacaaacattcacacatatgcac<br>atgcagtgaacacacacacacacacacacacacacacagagagagagaga<br>gagagagagagagagagagagagagagagagagagaagaagagagagagaga<br>aagaga
Dist MUT		atccaggtgtgtttgtaatcccagcactgaagaaatgAGCTACTAGCagaca<br>gggggaatctctagaattgctgggcagccagcctagctgaattggtgagatc

	taggctcagggagagactcagttacaaaagatagaaggaagagaaaccaagg aaggtactggatgtctagttagggatgcacacaaacattcacacatatgcac atgcagtgaacacacacacacacacacacgcacacacacagagagagagaga gagagagagagagagagagagagagagagagagaagaagagagagagaga aagaga
pT	tttttttcttttttcttttttcttttttttttttttttttttgcttgtctga atgaggc

GBlock sequences ordered. Capitalized sections represent sequences scrambled from the WT sequence.

Lentivirus production: Lentiviral particles were generated through transfection of HEK293T cells with lentiviral packaging plasmids and payload and lentiviral particles were titrated using Jurkat T cells as previously described (1) (Supplemental table 2).

Supplemental table 3: Third generation lentivirus transfection mix

Material	6 well format	10cm dish format
pMD2.G (aka VSVg)	625ng	3ug
pMDLg (aka pRRE)	312.5ng	1.5ug
pRSV-Rev	312.5ng	1.5ug
Lentiviral payload	1.25ug	6ug
Mirus TransIT-LT1	7.5uL	36uL
RPMI	200uL	1mL

Small molecule treatments:

MG132 treatment: Cells were treated with 10μM MG132 (Sigma-Aldrich: M8699) for four hours. After incubation, cells were brought to the microscope for imaging without changing the media.

$CoCl_2$ treatment: A stock solution of 1M was made using Cobalt(II) Chloride (Sigma; 232696-5G). The stock solutions were then filter sterilized using a PES 0.2µm syringe filter (Fisher scientific: 13100106) under a tissue culture hood, aliquoted in 500uL aliquots, and stored at -20°C.

For B16 cells, media was supplemented with 200µM $CoCl_2$. Cells are treated with $CoCl_2$ for 24 hours.

Puromycin selection: B16-tWt1-GFP cell lines were selected for using Puromycin. Puromycin stock solution was made from Puromycin-dihydrochloride (Wisent Bioproducts: 400-160-EM). Cells are transduced with lentivirus in a 24 well format as previously described. One day post transduction, they are passaged into one well of a 6 well plate. A well of untransduced cells of the same cell line is also seeded in one well of a 6 well format. Three days post transduction, media is replaced with media supplemented with 1µg/mL of Puromycin. Media was changed every other day with Puromycin supplemented media for six days, or until 100% of the untransduced control cells have died.

Cellular biology protocols:

Fluorescence Assisted Cell Sorting (FACS): FACS analyses were done on a ZE5 (Bio-Rad), CantoII (BD Biosciences), or an LSRII (BD Biosciecnes). All markers (eGFP, mCherry, Ametrine) were produced endogenously, and did not require antibody labeling. Intracellular Doxycycline levels were quantified using the 405nm laser and 525/50 detection filters. FACS data analyses and figure generation was done using FlowJo V10 (BD Life Sciences).

Single cell sorting was performed at the IRIC Flow Cytometry platform using the BD FACSAria III sorter. Each positive cell was sorted directly into a well of a 96 well flat bottom adherent plate containing 150uL of conditioned media (45% fresh cell media, 45% media used to grow the same cell line for 24 hours, and an additional 10% FBS).

Confocal microscopy & cell morphology picture: Cells were stained with Hoechst (2mg/mL) for 20 minutes, washed with PBS and then fresh DMEM free of phenol red was added prior to live cell imaging. All fluorescent microscopy images were taken using an LSM-880 (Zeiss). GFP was acquired using an Argon-488nm laser, and mCherry was acquired using an Argon-561nm laser. Images were processed using ImageJ. The cell morphology photos (**Fig.1E**) were taken using a white light Nikon Eclipse TS100 microscope, the 10x magnification objective lens, and a Nexus 5 smartphone.

Cell proliferation assay: B16-WT and B16-HG cells were seeded at equal density in triplicate. Every 24 hours, cells were harvested for counting using a Beckman Coulter cell counter, using the manufacturer's protocol.

Molecular Biology and Biochemical protocols:

Western blot: Proteins were prepared from cell RIPA cell lysate using the Wessel-Fluegge method. All Proteins were electrophoresed on pre-cast NuPage 4-12% Bis-Tris gel (Life Technologies: NP0321BOX), and migrated at 120V in MES buffer (Life Technologies: J62138.AP). Proteins were transferred to a methanol-soaked Polyvinylidene Fluoride (PVDF) membrane (Cytiva Life Sciences: 10600021) using a wet transfer box set to 200mA for 30 minutes in Towbin buffer. Primary antibody solutions are prepared in TBST (Tris-buffered saline with 0,5% tween) +3% BSA supplemented with 0.002% (g/ml) of sodium azide (Bioshop: SAZ001), and primary antibody. PVDF membrane sections were incubated in primary antibody solution at 4ºC overnight with rocking. Primary antibodies used were: anti-human HIF1α (1:1000, BD: 610958), anti-murine HIF1α (1:1000, Nocus Bio: nb100-134), GFP (1:1000, Invitrogen #A6455), Calnexin (1:3000, Enzo: ADI-SPA-860), Calreticulin (1:1000, BD Biosciences: 612136), E-Cadherin (1:1000, Cell Signaling Technology: 14472), N-Cadherin (1:1000, Cell Signaling Technology: 13116), Vimentin (1:1000, Santa Cruz Biotechnology: sc32322), WT1 (1:1000, RayBiotech: 102-11337). Images were processed using ImageJ (US National Institutes of Health).

Genomic PCR validation of canonical Wt1 exons: B16 genomic DNA was prepared using TriZol as per the manufacturer's protocol (ThermoFisher: 15596026). Oligo primers were designed to span complete exon sequences and intronic regions between them, or canonical *Wt1* promoter regions upstream of named exons (Supplemental table 3). PCRs were performed using DreamTaq (ThermoFisher: EP0701).

Supplemental table 3: Primers used for genomic PCR with expected product size

Wt1 Product	Forward Primer (5->3')	Reverse Primer (5'->3')	Size (bp)
prom. & E1	GAGAATCCGCAGGATCGCA	GCGTTGTCACTCGCATTTGA	674
prom. & E1a	CCCTGGTCCAGATCAACC	CCCTTCTCCATTCCTGAGTGG	511
E2 & E3	CAGGATACAGCACGGTCACT	CCTGCTGTAGGGCGTCC	571
E4 & E5	GTGGGTGTTTTTCACAGTGACA	TTGCTCTGCCCTTCTGTCC	1103

Quantitative real-time PCR (qPCR): RNA from cell pellets were generated using TriZol (ThermoFisher: 15596026) as per the manufacturers protocol. Reverse transcription was performed using the High Capacity cDNA Reverse Transcription Kit (Applied Biosystems) and 2ug of RNA. qPCR was performed using Advanced qPCR MasterMix HI-ROX (Wisent Bioproducts) as per the manufacturer's protocol. qPCR reactions were run in 10uL technical triplicates, in either 96 well or 384 well formats. Human melanoma qPCR data was acquired using biological duplicates. Ovarian cancer qPCR was acquired using technical triplicates. Primer sequences are detailed in Supplemental table 4.

Supplemental table 4: Primers used for qPCR

Gene	Forward (5'->3')	Reverse (5'->3')
18s rRNA	AGGAATTCCCAGTAAGTGCG	GCCTCACTAAACCATCCAA
hsa can. WT1	AGG CTT TGC TGC TGA GG	AGC ACA GGG TAC GAG AG
has tWT1	GAG CTG GTC TGA ACG AGA	AGC ACA GGG TAC GAG AG
RPL10	tcccacttgctgaaaaggtc	agcaggagcagctgtggt
TAGLN	cagtgtggccctgatgtg	caccagcttgctcagaatca
SNAI1	gagctgcaggactctaatcca	cggtggggttgaggatct
SNAI2	acagcgaactggacacacat	gatggggctgtatgctcct
CDH1	gaatgacaacaagcccgaat	gacctccatcacagaggttcc
CDH2	ctccatgtgccggatagc	cgatttcaccagaagcctctac
FN1	gaactatgatgccgaccagaa	ggttgtgcagatttcctcgt
ZEB1	cctaaaagagcacttaagaattcacag	catttcttactgcttatgtgtgagc
ZEB2	acaagccagggacagatca	gccacactctgtgcatttga

cDNA PCR: OVCAR3 RNA and cDNA were prepared using TriZol and High Capacity cDNA Reverse Transcription Kit as per the manufacturer's protocols (ThermoFisher & Applied Biosystems). G/P specific PCR products were generated using isoform specific forward primers (G: CCAACTATTTTTGTTCTCCACAGCAC; P: CCAAATGGCGACTGTGAGC) and a common reverse primer (GGACCGGGAGAACTTTCGC). PCR was performed using Pfusion as per the manufacturer's protocol (New England Biolabs: M0530S).

ChIP-qPCR analyses: B16-E7-WT1-GFP or B16wt cells were cultured following the LTHY protocol and treated with Dox once the cells were put into the 0.5% O_2 condition for 48 hours. Following this, cells are processed for ChIP as previously described (2). Briefly, cells are fixed with 1% formaldehyde for 10 minutes with shaking. Cross-linking is quenched with 150mM Glycine. Cells are then washed with PBS, collected by scrapping, and gently lysed with a 0.5% NP40 based buffer, which maintains nuclear membrane integrity. Nuclei are retained, then lysed with a 1% SDS buffer, and sonicated. Sonication was performed using a Bioruptor (Diagenode), 30 seconds ON, 30 seconds OFF at medium intensity for 10 total minutes at 4°C. DNA fragmentation quality is confirmed via agarose gel migration. 50µg of sonicated DNA is used per IP, diluted 1:20 with a dilution buffer and incubated overnight with 2.5µg of rabbit anti-GFP antibody (Invitrogen: #A6455) or Anti-HIF1α (Novus Bio nb100-134) with rotation at 4°C The following day, samples are incubated with Dynabeads (Thermofisher) for 2 hours at 4°C. After incubation, proteins are removed from the beads using a 1% SDS based buffer and incubation at 65°C. Proteins are then digested using Proteinase K (ThermoFisher EO0491) at 55°C for hour with shaking. DNA is finally purified using a DNA gel recovery kit (Zymo Research: D4001).

All probe sets are presented in Supplemental table 5. The negative control, *Kmda3,* and *Vegfa* probe sets were used as previously described; all other probe sets were designed by Primer-BLAST (3–5). The qPCR mixes, acquisition machine, and run settings were the same as for a regular cDNA qPCR run. Experiment was done with biological triplicates. Quantification was done using the fold-enrichment method (6).

Supplemental table 5: ChIP-qPCR probe sets.

Probe set	Forward (5'->3')	Reverse (5'->3')

Neg. ctrl. (*Fyn*)	ACAGGGGACTTAAAGGTGAGAGA	AGGCAAGCCATGCTTTGTTCTA
Kmd3a	GCAGCTCCATTCTTCCATTT	GCTCATGATCCTGGGTCTC
Car9	TCTACAAAAGGGCACTGTGAGT	CTGTGGACGGGCTGTACG
Vegfa	CGAGGGTTGGCGGCAGGAC	CAGTGGCGGGGAGTGAGACG
Wt1 int5 3.5	TGACTGTTCGGTTTTAAGGTCAGA	TGGTACCAGTTTCCTTTTACCATCT
Wt1 int5 7361	CATACCAGACACCAGGCACA	AATTAAACACGTGCGCCACC
Wt1 int5 7995	GTGTTCACGTGTGACAGCTCA	ATCGAGTCCTAGGGACTGAAGTTG
Wt1 int5 12.5k	TTTGAGTTCGTGGCTCTGCT	GGTTTCCAGAATGCAGTGGC
Wt1 int5 16kb	GAGAACCACACTGGGAAGCA	CAGTTGGCCCCTGCCTATTT
Wt1 HREs 18kb	TGGATGAGAGGAGCCACAGA	AGAATGCTGCAGGCTTCGAG

ChIPseq analyses: ChIPseq sample preparation was performed using the ChIP-qPCR sample preparation protocol with the following adjustment. Nuclear lysate Bioruptor sonication settings are increased to 1x12 minutes 30 seconds ON, 30 seconds OFF, Medium intensity to increase DNA fragmentation to the 200-600bp range. ChIP samples were submitted to the IRIC Genomics platform for library preparation using the KAPA library preparation kit and NGS on an Illumina NextSeq 500. Raw NGS data was analyzed by the IRIC Bioinformatics platform. Raw reads were trimmed using Trimmomatic, mapped to the murine genome (mm10) using BWA, and aligned reads were filtered using SAMtools (MAPQ > 20 & samflag 4) (7–9). Filtered read analysis was performed using MACS for peak calls and HOMER for functional annotation, known motif analyses, and *de novo* motif analysis (10,11).

Mapping of the WT1 motif to called peaks was done manually in Python using the Biopython module. Generation of gene coverage figures was done using the Spark python tool, using the following flags: -gs, -sm 10) (11. Annotation cluster bubbleplots were generated using DAVID functional annotation enrichment and rendered using Python (13,14).

Characterization of E7-WT1-GFP by Mass Spectrometry: HEK293T cells stably expressing pCW-E7K-tWt1-GFP were incubated with 2µg/mL Dox for 48 hours prior to lysis. Cells were lysed using the RIPA method as previously described, and (2,5µg) anti-GFP was used to IP the -GFP fusion protein using Protein A Sepharose beads. The IP sample was resuspended in 1X LDS

(Invitrogen) supplemented with 10mM DTT and run on a NuPage 4-12% Bis-Tris gel in MES buffer (Invitrogen). Following electrophoresis, bands corresponding to the expected size were excised from the gel and sent to the IRIC Proteomics platform digestion and analyses. The theoretical E7-WT1 protein sequence was used to search for peptide coverage, including post-translational modifications, using the search engine PEAKS Studio v10.5. Coverage of the tWT1-GFP CDS was calculated using Scaffold v4.8.3.

RNAseq runs & quantification: Frozen cell pellets were resuspended in QaiZol and total RNA was extracted using the Qaigen miRNeasy kit (Qiagen: 217084). mRNA library preparation and sequencing was performed by the McGill University and Genome Quebec Innovation Center using the KAPA rRNA-depleted (HMR) stranded library preparation for paired-end Illumina sequencing (Roche: 07962282001). Raw RNAseq reads were quality controlled using FASTQC (v0.11.5) on default settings (14). Reads were mapped to the murine genome (UCSC mm10) using Tophat (v2.1.1). Only reads with a single match to mm10 were kept for further analysis. Using the mouse genome reference annotation file (Mus_musculus.GRCm38.94.gtf), reads were counted on exons using coverageBed v2.24.0. Differential gene expression was calculated in R (v3.3.1) using DESeq2 (v1.14.1) and the Benjamini-Hochberg p-value adjustment (16).

Small RNA library preparation and next generation sequencing was performed by the McGill University and Genome Quebec Innovation Center. Library preparation was done using the NEB miRNA library preparation protocol (NEB: E7330S). miRNAseq was performed using single end 50bp reads on an Illumina HiSeq. Raw RNAseq reads were trimmed using cutadapt (v1.15) with options that favor specifity (--quality-cutoff 22,20 --error-rate 0.33 --overlap 2 --minimum-length 17 --maximum-length 30 --match-read-wildcards --trim-n) to maximize genomic mapping rate. Genomic mapping was done using miRDeep2 (v2.0.0.8) and bowtie1 (v1.2). Aside from the following options, all settings were set to default: reads shorter than 17nt are discarded; reads can map to up to ten places in the genome; at most; 1 mismatch is permitted per read. After genomic mapping, miRs are counted from the surviving reads. Differential expression was calculated using DESeq2 (v1.14.1) and the Benjamini-Hochberg p-value adjustment (16).

Additional bioinformatics analyses:

PCA analyses: Principle Component Analyses were performed using R and following the DESeq2 tutorial (16). The top 500 variable genes in the LTHY dataset were used as input.

RNAseq read-coverage plot: RNAseq read-coverage analyses were performed and rendered using IGV (17,18).

Transcription factor binding site analyses and scrambling: Transcription Factor Binding Site (TFBS) analyses were performed in R and used the following libraries: seqinr, TFBSTools (v1.10.0), Biostrings, JASPAR2018 (>=v1.0.0), ggbio, dplyr, GenomicRanges, tibble. The TFBS analysis table was used as input to a Python script generating histograms using the matplotlib module. Only transcription factors with a minimal expression of 100 averaged normalized DESeq2 reads in any condition, with a TFBS score of 0.95 or higher were considered. All listed transcription factors maintained expression above this cut-off at 0.5% O_2 and below.

TFBSs were scrambled using an in-house Python script, which partially uses published code for known motif analysis (19). Briefly, areas of interest are scrambled to a random sequence with equivalent GC content. Transcription factor binding sites overlapping or completely within the scrambled sequence are detected. Transcription factors which are not expressed in B16-HG cells are ignored. Scrambled sequences are manually modified until no transcription factor binding sites are detected.

Calculation of promoter activity: Geometric Mean Fluorescent Intensities (geoMFI) of ZsGreen for mCherry+ and mCherry- cells are calculated using the gates shown in **Fig.4C**. These values are used to make a ratio of ZsGreen geoMFI between transduced and untransduced cells and is calculated for both hypoxic and normoxic cells. For each construct, this ZsGreen ratio under hypoxia is normalized to its normoxic counterpart. This double normalized ZsGreen ratio is what’s presented in **Fig.4D-E**.

Sashimi plot generation: The WT1 RNAseq read location plot was generated using the sashimi-plot.py Python script from the ggsashimi project (20). Minimum read coverage was set to 3 to

remove primary transcript associated reads. Chromosomal range was set to chr:2 105162045-105174815. GRCm38.p6 was used for gene annotation.

Kozak strength evaluation: Kozak sequence scores were generated using the translation initiation site predictor tool developed by the Roos lab (21). Kozak similarity scores were rendered as a histogram using GraphPad v7.02.

Kmer-based identification of tWT1 isoforms: Tables of kmers for all TCGA samples were precomputed by the IRIC Bioinformatics platform using Jellyfish (22). Isoform specific mRNA junction sequences were used to quantify WT1 isoform level expression within the TCGA datasets using the km software and an implementation of the EM (Expectation-Maximization) algorithm that takes as input kmer counts for the sequences being quantified (in this case, P-tWT1 vs G-tWT1) (23). The following sequences were used to identify WT1 isoforms in TCGA expression data: canonical WT1 exon 1 (chr11:32435564-32434700), canonical WT1 exon 2 (chr11:32428619-32428497), canonical WT1 exon 1a (chr11:32430813-32430530), canonical WT1 exon 4 (chr11:32417654-32417577), a truncated version of isoform G exon 1 (chr11:32400146-32400351), and a truncated version of isoform G exon 2 (chr11:32399948-32400044). WT1 isoform level expression was quantified from the km runs using Python. A sample was considered to express an isoform if expression of the isoform specific junction was detected by km (i.e., all or most kmers for a given sequence are non-null). Isoform P expression was inferred by detection of the intronic sequence by km.

Survival curve generation: For the TCGA dataset, sets of patients expressing various WT1 isoforms were used to generate survival curves using Python scikit-survival, kaplan_meier_estimator function (v0.21.1).

Minimal post-median significance calculation for survival curves: Once a pair of survival curves are generated, the minimal median survival time is calculated. This is done by calculating the 50% survival time point for each curve, and taking the minimal date. Using that date, remove all data

used to generate the original survival curves that occur on or before that date. Then recalculate a 2-sided pvalue using these truncated datasets. This was accomplished in Python using the scikit-survival (v0.21.1) module, specifically the compare_survival function for significance calculations.

Sliding start point significance calculation for survival curves: The sliding starting point method is an extension of the minimal post-median method. Using a predefined date range, significance is calculated using data past each time point as previously described, and plotted against the date threshold used for the significance calculation. This was accomplished in Python using the scikit-survival module for calculation of significance, and matplotlib for graphics.

Leucegene dataset: The current sample cohort consists of 529 primary samples acquired and managed by the BCLQ among which 452 are AML samples with 373 samples classified as diagnostic AML samples (excluding acute promyelocytic leukemia (APL). Leucegene transcriptomic data was also deposited in the Gene Expression Omnibus (GEO). Super series datasets (GSE67040, GSE48173), subseries datasets (GSE52656, GSE48846, GSE98310, GSE49642, GSE62190, GSE67039, GSE66917, GSE106272, GSE51984, GSE49601, GSE94339). Data availability can be obtained here: https://data.leucegene.iric.ca/about?lang=en.

Supplemental Materials and Methods References:

1. Connolly, A, Rebecca Panes, Margaux Tual, Raphael Lafortune, Angelique Bellemare-Pelletier, Etienne Gagnon. TMEM16F mediates bystander TCR-CD3 membrane dissociation at the immunological synapse and potentiates T cell activation. Sci Signal 14, eabb5146 (2021).

2. Arnold PK, Jackson BT, Paras KI, Brunner JS, Hart ML, Newsom OJ, et al. A non-canonical tricarboxylic acid cycle underlies cellular identity. Nature. 2022 Mar;603(7901):477–81.

3. Kann M, Ettou S, Jung YL, Lenz MO, Taglienti ME, Park PJ, et al. Genome-Wide Analysis of Wilms' Tumor 1-Controlled Gene Expression in Podocytes Reveals Key Regulatory Mechanisms. JASN. 2015 Sep 1;26(9):2097–104.

4. He Q, Gao Z, Yin J, Zhang J, Yun Z, Ye J. Regulation of HIF-1{alpha} activity in adipose tissue by obesity-associated factors: adipogenesis, insulin, and hypoxia. Am J Physiol Endocrinol Metab. 2011 May;300(5):E877-885.

5. Ye J, Coulouris G, Zaretskaya I, Cutcutache I, Rozen S, Madden TL. Primer-BLAST: a tool to design target-specific primers for polymerase chain reaction. BMC Bioinformatics. 2012 Jun 18;13:134.

6. Lacazette E. A laboratory practical illustrating the use of the ChIP-qPCR method in a robust model: Estrogen receptor alpha immunoprecipitation using Mcf-7 culture cells. Biochemistry and Molecular Biology Education. 2017;45(2):152–60.

7. Bolger AM, Lohse M, Usadel B. Trimmomatic: a flexible trimmer for Illumina sequence data. Bioinformatics. 2014 Aug 1;30(15):2114–20.

8. Li H, Durbin R. Fast and accurate short read alignment with Burrows-Wheeler transform. Bioinformatics. 2009 Jul 15;25(14):1754–60.

9. Li H, Handsaker B, Wysoker A, Fennell T, Ruan J, Homer N, et al. The Sequence Alignment/Map format and SAMtools. Bioinformatics. 2009 Aug 15;25(16):2078–9.

10. Zhang Y, Liu T, Meyer CA, Eeckhoute J, Johnson DS, Bernstein BE, et al. Model-based Analysis of ChIP-Seq (MACS). Genome Biology. 2008 Sep 17;9(9):R137.

11. Heinz S, Benner C, Spann N, Bertolino E, Lin YC, Laslo P, et al. Simple combinations of lineage-determining transcription factors prime cis-regulatory elements required for macrophage and B cell identities. Mol Cell. 2010 May 28;38(4):576–89.

12. Kurtenbach S, Harbour JW. SparK: A Publication-quality NGS Visualization Tool [Internet]. bioRxiv; 2019 [cited 2022 Aug 29]. p. 845529. Available from: https://www.biorxiv.org/content/10.1101/845529v1

13. Huang DW, Sherman BT, Lempicki RA. Systematic and integrative analysis of large gene lists using DAVID bioinformatics resources. Nat Protoc. 2009;4(1):44–57.

14. Sherman BT, Hao M, Qiu J, Jiao X, Baseler MW, Lane HC, et al. DAVID: a web server for functional enrichment analysis and functional annotation of gene lists (2021 update). Nucleic Acids Res. 2022 Mar 23;gkac194.

15. Andrews S. FastQC: A Quality Control Tool for High Throughput Sequence Data [Online]. [Internet]. Available from: http://www.bioinformatics.babraham.ac.uk/projects/fastqc/

16. Love MI, Huber W, Anders S. Moderated estimation of fold change and dispersion for RNA-seq data with DESeq2. Genome Biol. 2014;15(12):550.

17. Robinson JT, Thorvaldsdóttir H, Winckler W, Guttman M, Lander ES, Getz G, et al. Integrative genomics viewer. Nat Biotechnol. 2011 Jan;29(1):24–6.

18. Robinson JT, Thorvaldsdóttir H, Wenger AM, Zehir A, Mesirov JP. Variant Review with the Integrative Genomics Viewer. Cancer Research. 2017 Oct 31;77(21):e31–4.

19. Oguztuzun C, Yasar P, Yavuz K, Muyan M, Can T. MotifGenie: a Python application for searching transcription factor binding sequences using ChIP-Seq datasets. Bioinformatics. 2021 Nov 18;37(22):4238–9.

20. Garrido-Martín D, Palumbo E, Guigó R, Breschi A. ggsashimi: Sashimi plot revised for browser- and annotation-independent splicing visualization. PLOS Computational Biology. 2018 Aug 17;14(8):e1006360.

21. Gleason AC, Ghadge G, Chen J, Sonobe Y, Roos RP. Machine learning predicts translation initiation sites in neurologic diseases with nucleotide repeat expansions. PLOS ONE. 2022 Jun 1;17(6):e0256411.

22. Marçais G, Kingsford C. A fast, lock-free approach for efficient parallel counting of occurrences of k-mers. Bioinformatics. 2011 Mar 15;27(6):764–70.

23. Audemard EO, Gendron P, Feghaly A, Lavallée VP, Hébert J, Sauvageau G, et al. Targeted variant detection using unaligned RNA-Seq reads. Life Sci Alliance. 2019 Aug;2(4):e201900336.
