# Supplementary material for: Long-term severe hypoxia adaptation induces non-canonical EMT and a novel Wilms Tumor 1 (WT1) isoform
Source: Cancer Gene Ther. 2024 Jul 8;31(8):1237–50. doi: 10.1038/s41417-024-00795-3 (PMC11327107; doi:10.1038/s41417-024-00795-3)
Supplement: Supplementary file 2 — Supplemental figures [file 41417_2024_795_MOESM2_ESM.pdf]

Figure S1

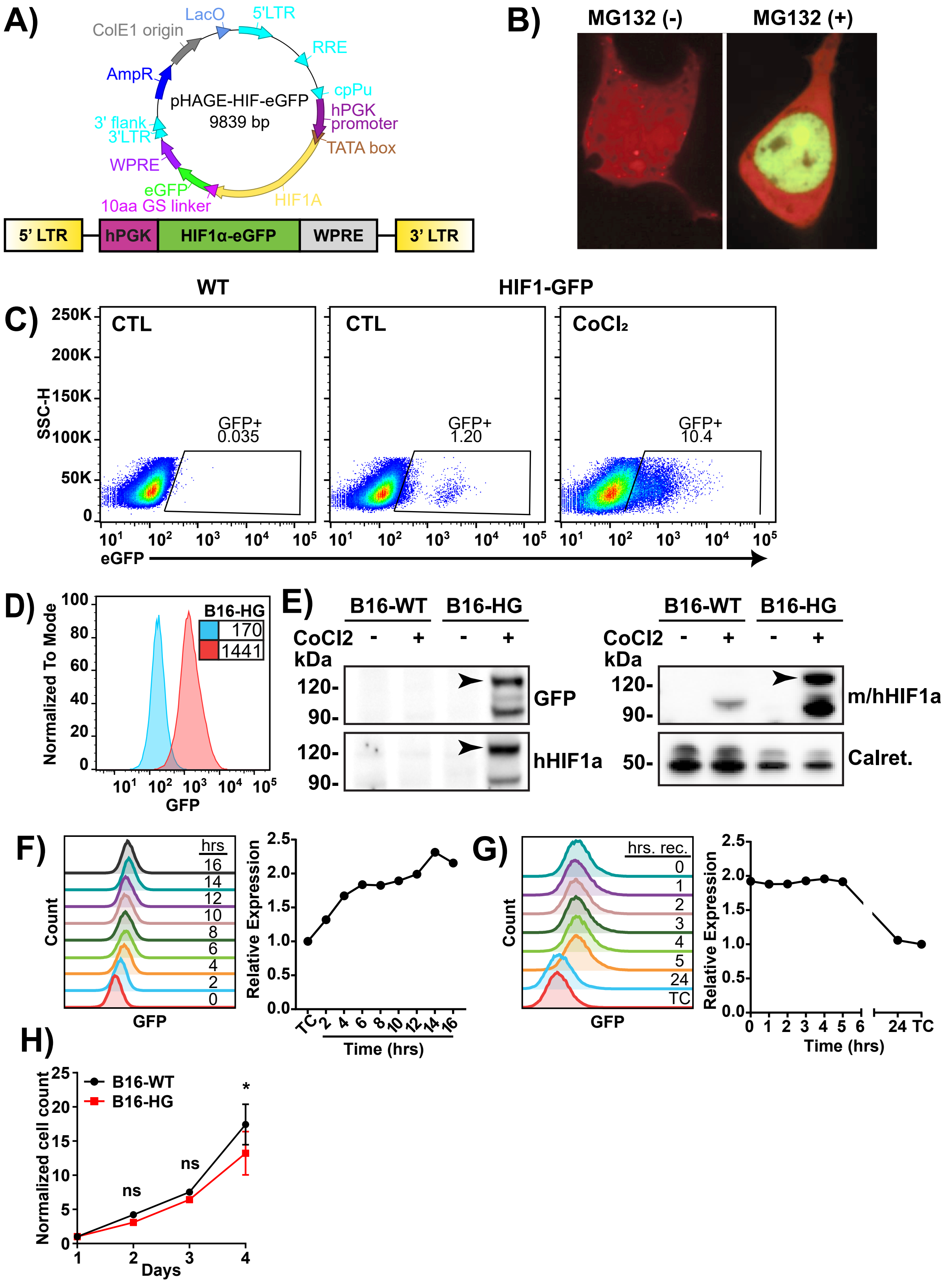

A)
ColE1 origin
LacO
5'LTR
RRE
AmpR
cpPu
pHAGE-HIF-eGFP
9839 bp
hPGK
promoter
3' flank
3'LTR
TATA box
WPRE
eGFP
HIF1A
10aa GS linker
5' LTR
hPGK
HIF1α-eGFP
WPRE
3' LTR
B)
MG132 (-)
MG132 (+)
C)
WT
HIF1-GFP
CTL
CTL
CoCl₂
SSC-H
GFP+
0.035
GFP+
1.20
GFP+
10.4
eGFP
D)
Normalized To Mode
B16-HG
170
1441
GFP
E)
B16-WT
B16-HG
CoCl2
kDa
GFP
hHIF1a
m/hHIF1a
Calret.
F)
hrs
Count
GFP
Relative Expression
Time (hrs)
G)
hrs. rec.
Count
GFP
Relative Expression
Time (hrs)
H)
B16-WT
B16-HG
Normalized cell count
ns
ns
Days

Supplemental 1: A) Top: Lentiviral plasmid map for pHAGE-HIF1α-eGFP. HPGK: Human *PGK* promoter. WPRE: Woodchuck hepatitis virus Post-transcriptional Regulatory Element. Bottom: Lentiviral payload. **B)** HEK cells transfected with the HIF1α-eGFP hypoxia reporter, 40hrs post-transfection. Left: Cells under standard TC conditions. Right: Cells treated with 10μM MG132 for four hours. Green: HIF1α-GFP. Red: mCherry (cytoplasmic). **C)** FACS plot of B16-WT cells (left), transduced B16-HIF1α-eGFP cells cultured without (middle) or with 200μM $CoCl_2$ (right) for 24 hours. **D)** GFP fluorescence intensities of most dynamic B16-HIF1α-eGFP clone. Blue: cells under standard TC conditions. Red: cells 24hrs after addition of 200μM $CoCl_2$ to cell media. Numbers represent geometric Mean Fluorescent Intensity (geoMFI) of GFP signal. **E)** Western blot of HIF1α-eGFP fusion protein in clone B16-HG cells without and with 200μM $CoCl_2$ treatments for 24hrs. The membranes were probed with either anti-GFP (GFP), anti-human specific HIF1α (hHIF1α), or anti-mouse and human specific HIF1α (m/hHIF1α). Anti-calreticulin was used as loading control. Arrowhead indicates full-length hHIF1α-GFP, and the asterisk indicates endogenous murine HIFα. **F)** Induction kinetics of the B16-HG cell line. Cells were incubated at 0.2% O_2 for the indicated time. Normoxic media was replaced with hypoxic media at the beginning of the assay. After the indicated incubation time, cells were processed by FACS. Listed numbers are the GFP geoMFI. **G)** HIF1α-eGFP degradation kinetics following 48hrs of incubation at 1% O_2. Following extraction from the hypoxia incubation chamber, hypoxic media was changed for normoxic media. Following the indicated recovery time, cells were processed by FACS. **H)** Comparative proliferation between B16-WT and B16-HG cells.

Figure S2

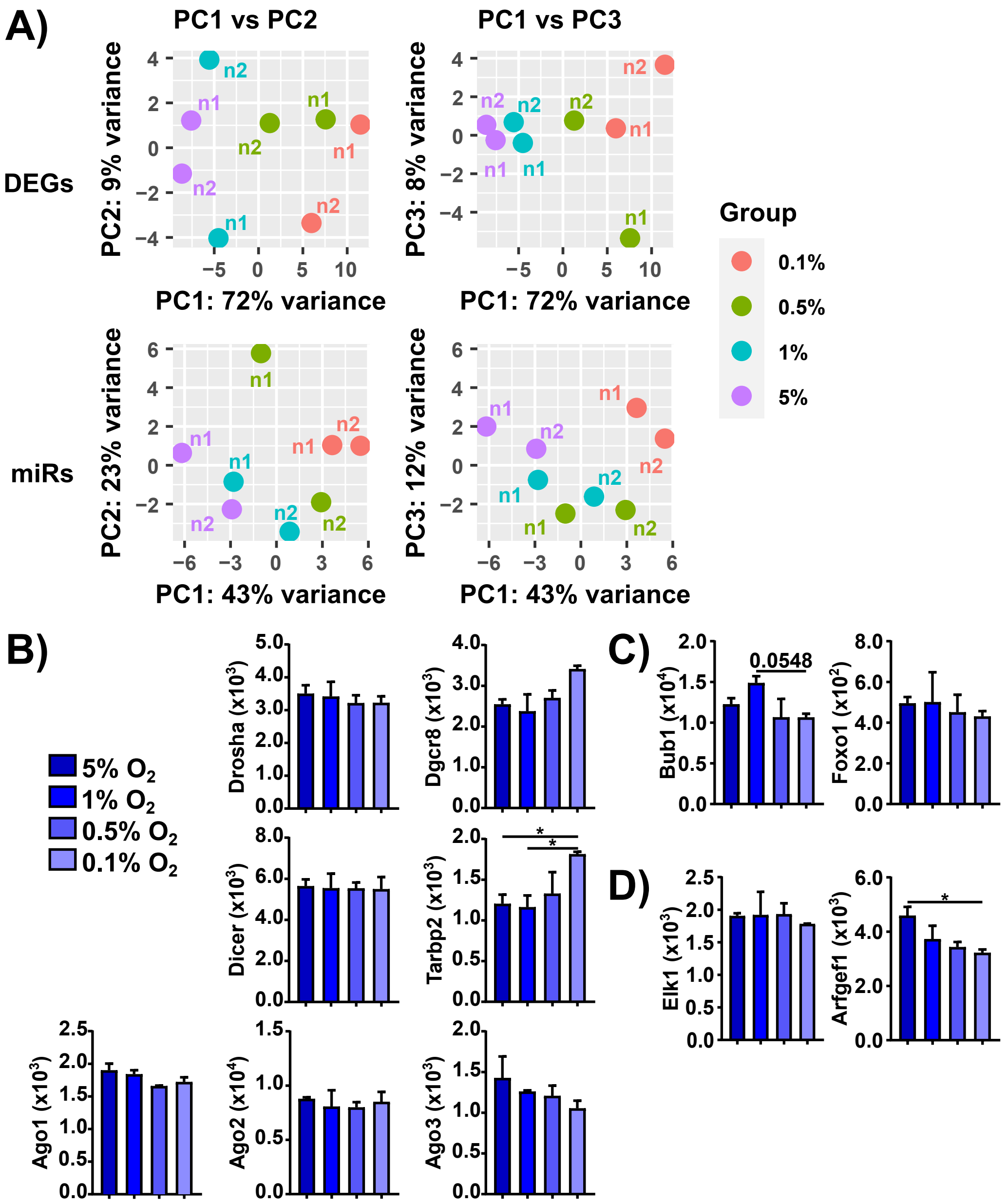

A)
PC1 vs PC2
PC1 vs PC3
DEGs
miRs
PC2: 9% variance
PC3: 8% variance
PC1: 72% variance
PC2: 23% variance
PC3: 12% variance
PC1: 43% variance
Group
0.1%
0.5%
1%
5%
B)
5% O2
1% O2
0.5% O2
0.1% O2
Drosha (x10^3)
Dgcr8 (x10^3)
Dicer (x10^3)
Tarbp2 (x10^3)
Ago1 (x10^3)
Ago2 (x10^4)
Ago3 (x10^3)
C)
Bub1 (x10^4)
0.0548
Foxo1 (x10^2)
D)
Elk1 (x10^3)
Arfgef1 (x10^3)

Supplemental 2: **A)** PCA analyses of LTHY mRNAseq dataset (top) and LTHY miRNAseq dataset (bottom). **B)** Expression of miR biogenesis genes in the LTHY dataset. **C)** Expression levels of literature established miR-27a targets. **D)** Expression levels of literature established miR-27b targets. **B-D)** Values are DESeq2 normalized reads, error bars are SD. * denotes relative significance as calculated by DESeq2 Benjamini-Hochberg adjusted p-value (padj). *: padj < 0.05, **: padj < 0.01, ***: padj < 0.001.

Figure S3

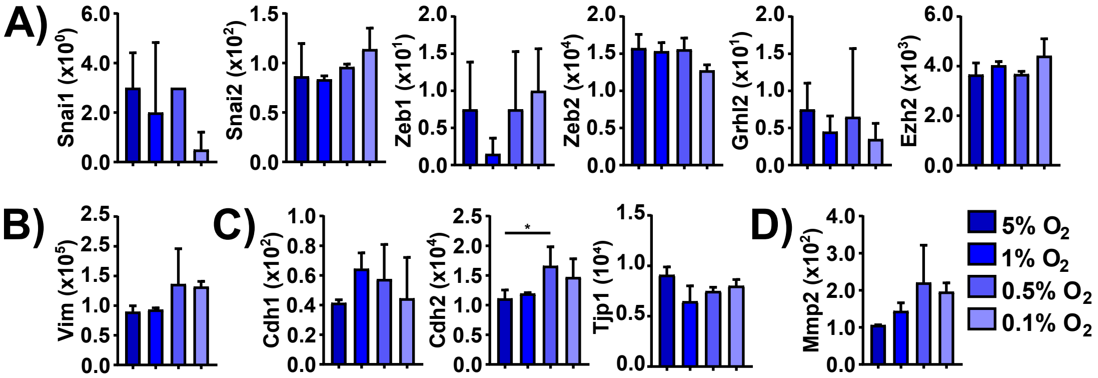

A)
Snai1 ($x10^0$)
Snai2 ($x10^2$)
Zeb1 ($x10^1$)
Zeb2 ($x10^4$)
Grhl2 ($x10^1$)
Ezh2 ($x10^3$)
B)
Vim ($x10^5$)
C)
Cdh1 ($x10^2$)
Cdh2 ($x10^4$)
*
Tjp1 (10^4)
D)
Mmp2 ($x10^2$)
5% O_2
1% O_2
0.5% O_2
0.1% O_2

Supplemental 3: **A)** Expressions of canonical EMT driving genes. Data not shown for genes with zero expression. **B)** Expression profile for Vimentin. **C)** Expression profiles of E-cadherin (Cdh1) N-cadherin (Cdh2), and ZO-1 (Tjp1). **D)** Expression profile for Mmp2. **A-D)** Values are DESeq2 normalized reads, error bars are SD. * denotes relative significance as calculated by DESeq2 Benjamini-Hochberg adjusted p-value (padj). *: padj < 0.05, **: padj < 0.01, ***: padj < 0.001.

Figue S4

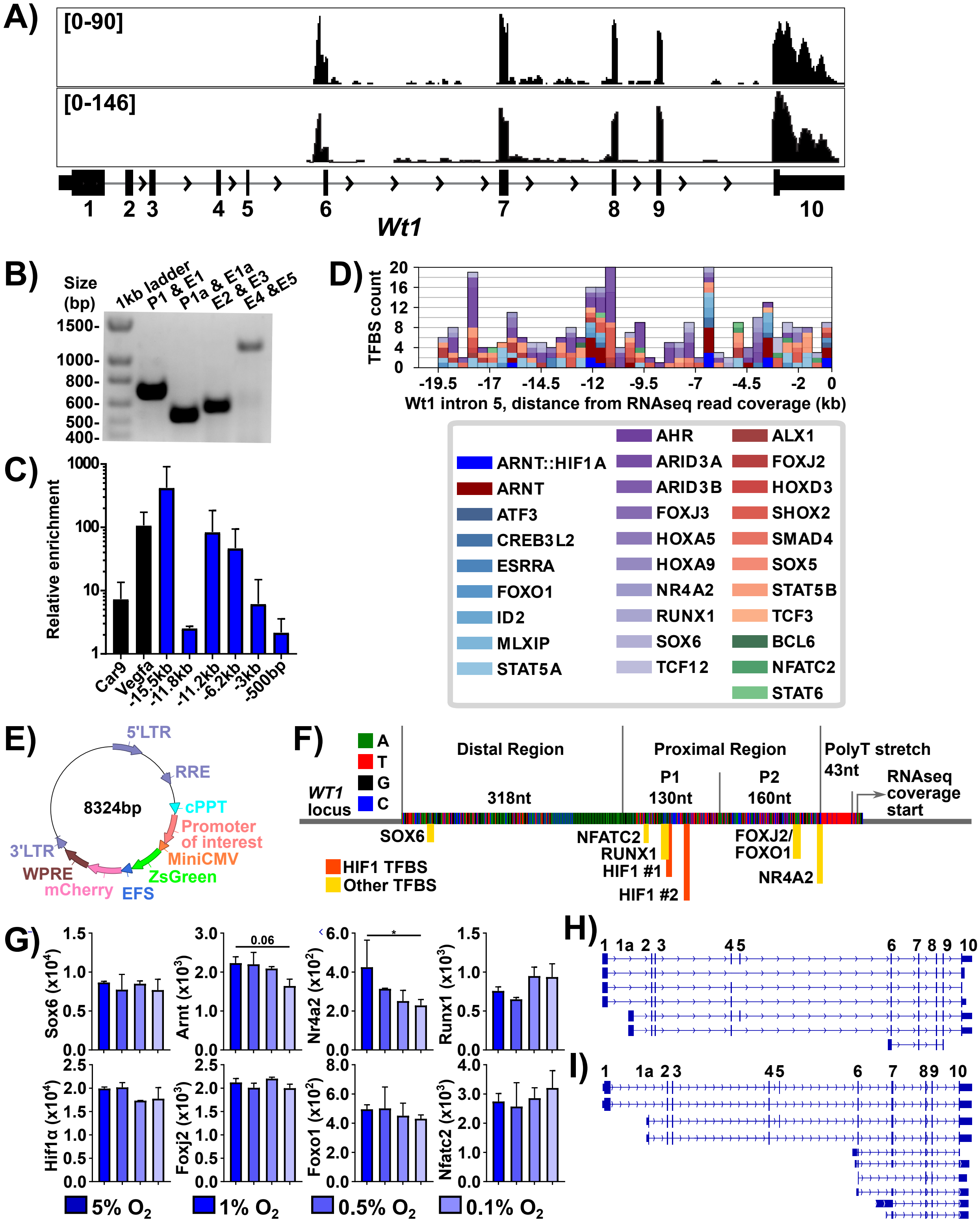

A)
[0-90]
[0-146]
1 2 3 4 5 6 7 8 9 10
Wt1
B)
Size (bp)
1kb ladder
P1 & E1
P1a & E1a
E2 & E3
E4 &E5
1500-
1000-
800-
600-
500-
400-
C)
Relative enrichment
1000
100
10
1
Car9
Vegfa
-15.5kb
-11.8kb
-11.2kb
-6.2kb
-3kb
-500bp
D)
TFBS count
Wt1 intron 5, distance from RNAseq read coverage (kb)
ARNT::HIF1A
ARNT
ATF3
CREB3L2
ESRRA
FOXO1
ID2
MLXIP
STAT5A
AHR
ARID3A
ARID3B
FOXJ3
HOXA5
HOXA9
NR4A2
RUNX1
SOX6
TCF12
ALX1
FOXJ2
HOXD3
SHOX2
SMAD4
SOX5
STAT5B
TCF3
BCL6
NFATC2
STAT6
E)
5'LTR
RRE
cPPT
Promoter of interest
MiniCMV
ZsGreen
EFS
mCherry
WPRE
3'LTR
8324bp
F)
A
T
G
C
WT1 locus
Distal Region
318nt
Proximal Region
P1 130nt
P2 160nt
PolyT stretch 43nt
RNAseq coverage start
SOX6
NFATC2
RUNX1
HIF1 #1
HIF1 #2
FOXJ2/ FOXO1
NR4A2
HIF1 TFBS
Other TFBS
G)
Sox6 (x10⁴)
Arnt (x10³)
0.06
Nr4a2 (x10²)
*
Runx1 (x10³)
Hif1α (x10⁴)
Foxj2 (x10³)
Foxo1 (x10²)
Nfatc2 (x10³)
5% O2
1% O2
0.5% O2
0.1% O2
H)
1 1a 2 3 4 5 6 7 8 9 10
I)
1 1a 2 3 4 5 6 7 8 9 10

Figure 4 supplemental: A) LTHY read coverage of the Wt1 locus, for both replicates at 0.1% O_2 of the LTHY time course, generated in IGV. Number ranges are coverage depths at the individual nucleotide level. Introns 1-4 are condensed for visual clarity. **B)** Genomic PCR products for B16-HG Wt1 exons 1-5. Lanes from left to right: 1kb ladder, exon 1 + promoter, exon 1a + promoter, exons 2&3, exons 4&5. **C)** HIF1α-ChIP-qPCR. B16-HG cells underwent the LTHY protocol up to 36hrs of exposure at 0.5% O_2, and were then processed for ChIP-qPCR. Negative control used was Fyn_neg probe. Experiment represents biological triplicates, error bars are SD. Black: positive controls, ChIP-qPCR for promoter regions of canonical hypoxia-senstive genes *Car9* and *Vegfa*. **D)** Transcription Factor Binding Site analysis of murine *Wt1* intron 5 from beginning of intron 5 to beginning of RNAseq read coverage for Wt1. Only considered TFBSs with a score ≥ 0.95. Intron 5 sequence is broken into 40 bins, ~500bp/bin. Colored TFs have a minimal expression of ≥ 100 DESeq2 normalized reads across any LTHY condition. Analysis done use TFBStools in R. **E)** Plasmid map for lentiviral promoter reporter system. LTR: Long Terminal Repeat. RRE: REV response element. EFS: EF1-alpha short promoter. WPRE: Woodchuck Hepatitis Virus (WHV) Posttranscriptional Regulatory Element. **F)** Diagram of genomic region used in hypoxic promoter reporter constructs. The 651nt region immediately upstream of the beginning of the WT1 RNAseq coverage is broken into four subregions. Top enriched TFBSs of TFs expressed in the RNAseq dataset are displayed. **G)** Expression profiles for transcription factors of interest across the LTHY time course. Values are DESeq2 normalized reads, error bars are SD. *: $p_{adj} < 0.05$. **H)** Known murine (GRCm39) Wt1 mRNA isoforms. **I)** Known human (GRCh38) WT1 mRNA isoforms.

Figure S5

A)

```
  1 gaatgaggcacacagcaccagacagtttgcctctgagggtgaattttggggcaggggtggctgcaaagtggtaacacacagacggaatggcctcggactt
     E * G T Q H Q T V C L * G * I L G Q G W L Q S G N T Q T E W P R T
      N E A H S T R Q F A S E G E F W G R G G C K V V T H R R N G L G L
       M R H T A P D S L P L R V N F G A G V A A K W * H T D G M A S D F
101 cacagagccctgttccagagtgagcccatggagtgagGTAAGACTCTGTATTTCCAAAAGGTGACCATGAACCAACTTTAACCCTTTGTTTGCAGccacg
    S Q S P V P E * A H G V R * D S V F P K G D H E P T L T L C L Q P R
     H R A L F Q S E P M E * G K T L Y F Q K V T M N Q L * P F V C S H
      T E P C S R V S P W S E V R L C I S K R * P * T N F N P L F A A T
201 gcacagggtatgagagtgagaaccacacggcccccatcctctgtggtgcccagtacagaatacacacccacggggtcttccgaggcattcaggatgtgcg
      H R V * E * E P H G P H P L W C P V Q N T H P R G L P R H S G C A
    G T G Y E S E N H T A P I L C G A Q Y R I H T H G V F R G I Q D V R
     A Q G M R V R T T R P P S S V V P S T E Y T P T G S S E A F R M C
```

B)

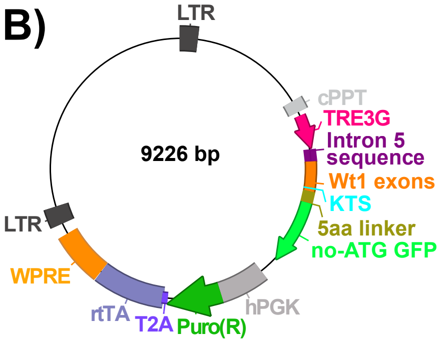

C)

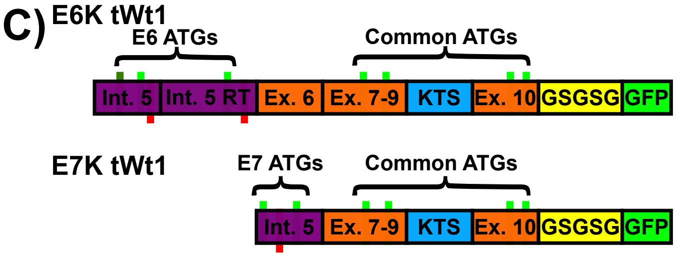

D)

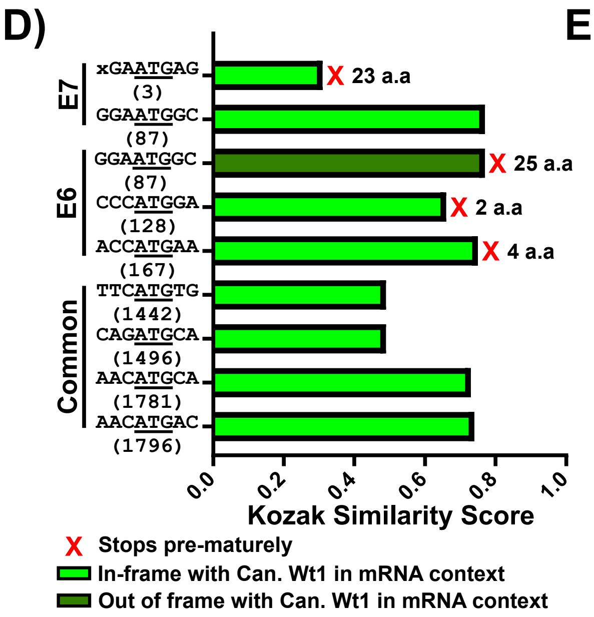

E)

Peptide sequence	Peptide start index	Best Peptide identification probability
ASDFTEPCSR	2	99.70%
SDFTEPCSR	3	99.70%
VSPWSEDVRR	12	99.70%
RVSGVAPTLVR	21	99.70%
VSGVAPTLVR	22	99.70%
SASETSEKRPF	32	99.60%
SASETSEKRPFMCAYPGCNKR	32	99.70%
AYPGCNKR	45	96.40%
YFKLSHLQMHSR	53	99.70%
LSHLQMHSR	56	99.70%
SHLQMHSR	57	99.00%
KHTGEKPYQCDFKDCER	65	99.70%
KHTGEKPYQCDFKDCERR	65	99.70%
FSRSDQLKR	83	99.70%
HTGVKPFQCK	96	99.70%
SDHLKTHTR	114	99.70%
THTGKTSEKPFSCR	123	99.70%
TSEKPFSCR	128	99.70%
FARSDELVR	144	99.70%
SDELVRHHNMHQR	147	99.70%
SAMPEGYVQER	259	99.70%
TIFFKDDGNYKTR	270	99.70%

Figure 5 Supplemental: A) Potential open reading frames derived from the tWt1 intron 5 sequence in E6 isoforms. Purple: Intron 5 derived sequence. Uppercase section is E6 specific read-through event. Orange: Exon 7 derived sequence. Bold: Canonical WT1 ORF (second ORF). Bright-green/dark-green: In/out of frame start codons. Red: Stop codons. **B)** Plasmid map for lentiviral Doxycycline-inducible expression plasmids. LTR: Long Terminal Repeat. cPPT: central polypurine tract. hPGK: human Phosphyglycerate Kinae 1 promoter. rtTA: reverse tetracycline-controlled transactivator. WPRE: Woodchuck Hepatitis Virus (WHV) Posttranscriptional Regulatory Element. Note the GFP lacks the start codon. **C)** tWt1-GFP isoform coding sequences. Purple: Intron 5 sequence. Orange: Exonic sequence. Yellow: Flexible linker, with peptide sequence. Green: GFP CDS, lacking the start codon. Green boxes above coding sequence are start codons; Bright-green are in-frame, dark green are out of frame. Red boxes below coding sequence are in-frame stop codons. RT: Read-Through. **D)** Kozak similarity scores for start codons of E7 tWt1 mRNA. Bright green represents start codons in-frame with the canonical Wt1 CDS. Dark green are out of frame start codons. Numbers beneath kozak sequence represent start codon position in transcript. E6 and E7 positions are relative to the E6 sequence. Common start codons are relative to NM_144783.2. **E)** Table of peptides identified in Mass spectrometry analysis of E7-K GFP fusion protein. Peptides were mapped to the theoretical E7-K peptide sequence. Search settings: PEAKS Studio v10.5; fragment tolerance: 10.0 PPM; fixed modifications: +57 on C (carbamidomethyl); variable modifications: +1 on NQ (Deamidated), +16 on M (Oxidation), +42 on n (Acetyl), +80 on STY (Phospho); digestion enzyme: Trypsin. Peptide coverage of the E7K CDS was calculated using Scaffold v4.8.3.

Figure S6

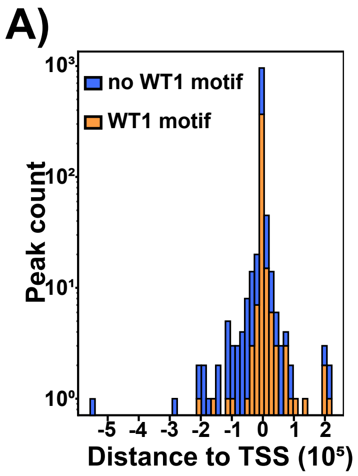

A)
no WT1 motif
WT1 motif
Peak count
10^3
10^2
10^1
10^0
-5 -4 -3 -2 -1 0 1 2
Distance to TSS (10^5)

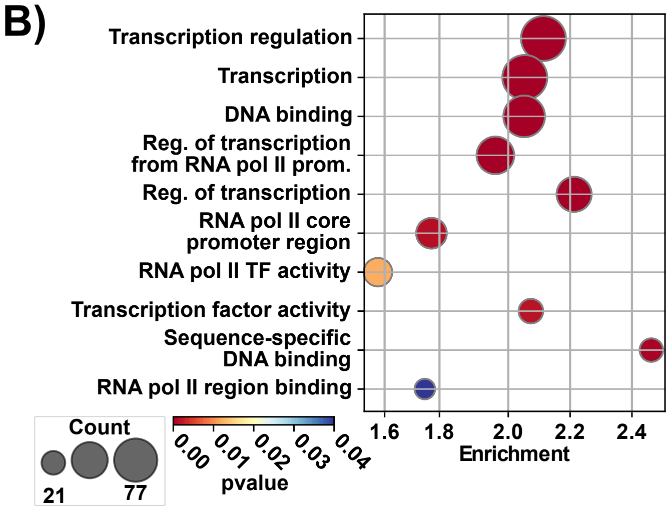

B)
Transcription regulation
Transcription
DNA binding
Reg. of transcription from RNA pol II prom.
Reg. of transcription
RNA pol II core promoter region
RNA pol II TF activity
Transcription factor activity
Sequence-specific DNA binding
RNA pol II region binding
Count
21
77
0.00 0.01 0.02 0.03 0.04
pvalue
1.6 1.8 2.0 2.2 2.4
Enrichment

Figure 6 supplemental: **A)** Distribution of E7-tWt1 ChIPSeq peak locations from nearest gene TSS. Motif analysis was performed using an in-house analysis pipeline, using the canonical WT1 DNA binding motif. **B)** GO term annotation bubbleplot of genes from E7-tWt1 ChIPSeq peaks containing the canonical WT1 motif.

Figure S7

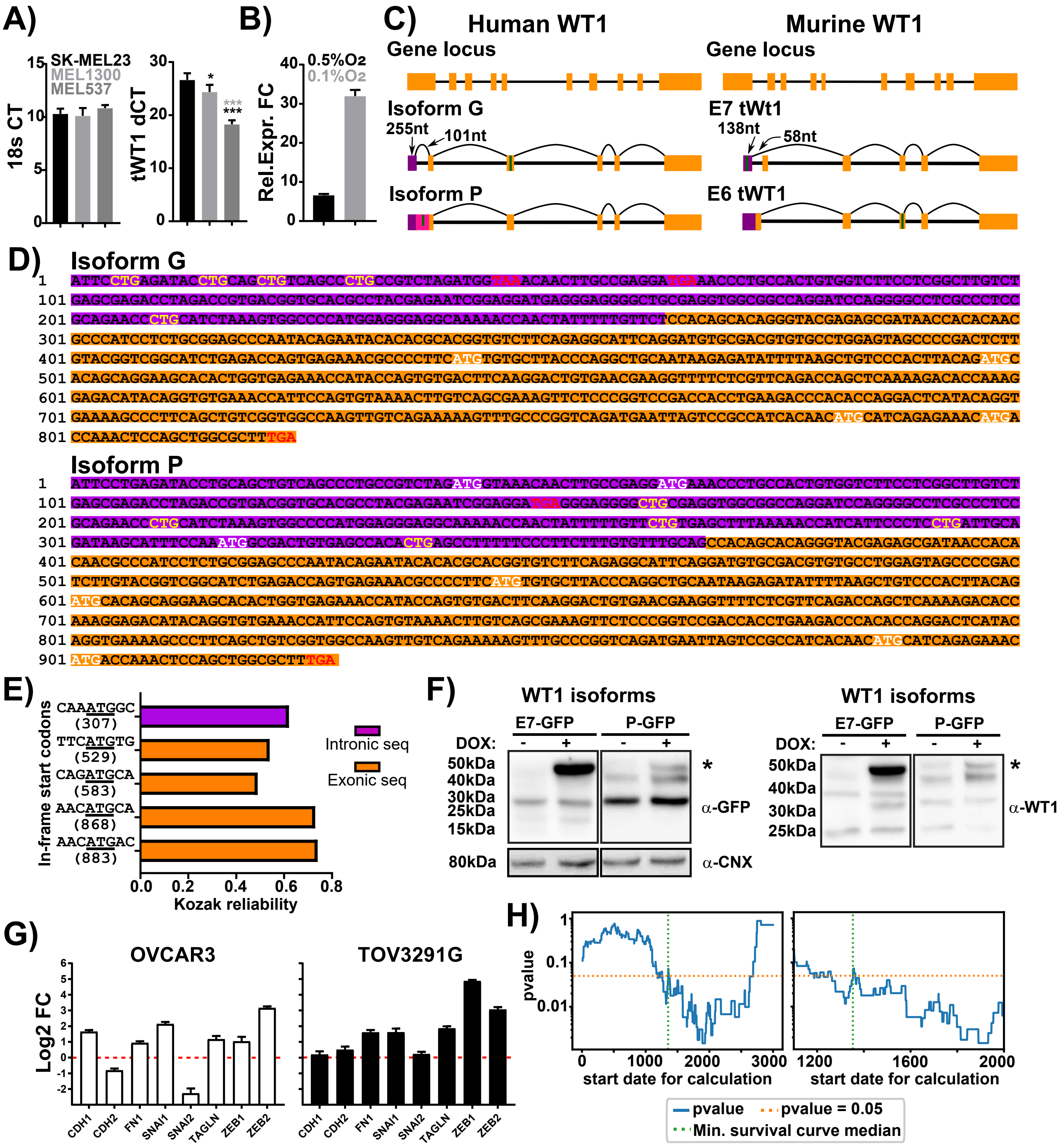
A)
SK-MEL23
MEL1300
MEL537
18s CT
tWT1 dCT
*
***
***
B)
0.5%O2
0.1%O2
Rel.Expr. FC
C)
Human WT1
Gene locus
Isoform G
255nt
101nt
Isoform P
Murine WT1
Gene locus
E7 tWt1
138nt
58nt
E6 tWT1
D)
Isoform G
1 ATTCCTGAGATACCTGCAGCTGTCAGCCCTGCCGTCTAGATGGTAAACAACTTGCCGAGGATGAAACCCTGCCACTGTGGTCTTCCTCGGCTTGTCT
101 GAGCGAGACCTAGACCGTGACGGTGCACGCCTACGAGAATCGGAGGATGAGGGAGGGGCTGCGAGGTGGCGGCCAGGATCCAGGGGCCTCGCCCTCC
201 GCAGAACCCTGCATCTAAAGTGGCCCCATGGAGGGAGGCAAAAACCAACTATTTTTGTTCTCCACAGCACAGGGTACGAGAGCGATAACCACACAAC
301 GCCCATCCTCTGCGGAGCCCAATACAGAATACACACGCACGGTGTCTTCAGAGGCATTCAGGATGTGCGACGTGTGCCTGGAGTAGCCCCGACTCTT
401 GTACGGTCGGCATCTGAGACCAGTGAGAAACGCCCCTTCATGTGTGCTTACCCAGGCTGCAATAAGAGATATTTTAAGCTGTCCCACTTACAGATGC
501 ACAGCAGGAAGCACACTGGTGAGAAACCATACCAGTGTGACTTCAAGGACTGTGAACGAAGGTTTTCTCGTTCAGACCAGCTCAAAAGACACCAAAG
601 GAGACATACAGGTGTGAAACCATTCCAGTGTAAAACTTGTCAGCGAAAGTTCTCCCGGTCCGACCACCTGAAGACCCACACCAGGACTCATACAGGT
701 GAAAAGCCCTTCAGCTGTCGGTGGCCAAGTTGTCAGAAAAAGTTTGCCCGGTCAGATGAATTAGTCCGCCATCACAACATGCATCAGAGAAACATGA
801 CCAAACTCCAGCTGGCGCTTTGA
Isoform P
1 ATTCCTGAGATACCTGCAGCTGTCAGCCCTGCCGTCTAGATGGTAAACAACTTGCCGAGGATGAAACCCTGCCACTGTGGTCTTCCTCGGCTTGTCT
101 GAGCGAGACCTAGACCGTGACGGTGCACGCCTACGAGAATCGGAGGATGAGGGAGGGGCTGCGAGGTGGCGGCCAGGATCCAGGGGCCTCGCCCTCC
201 GCAGAACCCTGCATCTAAAGTGGCCCCATGGAGGGAGGCAAAAACCAACTATTTTTGTTCTGTGAGCTTTAAAAACCATCATTCCCTCCTGATTGCA
301 GATAAGCATTTCCAAATGGCGACTGTGAGCCACACTGAGCCTTTTTCCCTTCTTTGTGTTTGCAGCCACAGCACAGGGTACGAGAGCGATAACCACA
401 CAACGCCCATCCTCTGCGGAGCCCAATACAGAATACACACGCACGGTGTCTTCAGAGGCATTCAGGATGTGCGACGTGTGCCTGGAGTAGCCCCGAC
501 TCTTGTACGGTCGGCATCTGAGACCAGTGAGAAACGCCCCTTCATGTGTGCTTACCCAGGCTGCAATAAGAGATATTTTAAGCTGTCCCACTTACAG
601 ATGCACAGCAGGAAGCACACTGGTGAGAAACCATACCAGTGTGACTTCAAGGACTGTGAACGAAGGTTTTCTCGTTCAGACCAGCTCAAAAGACACC
701 AAAGGAGACATACAGGTGTGAAACCATTCCAGTGTAAAACTTGTCAGCGAAAGTTCTCCCGGTCCGACCACCTGAAGACCCACACCAGGACTCATAC
801 AGGTGAAAAGCCCTTCAGCTGTCGGTGGCCAAGTTGTCAGAAAAAGTTTGCCCGGTCAGATGAATTAGTCCGCCATCACAACATGCATCAGAGAAAC
901 ATGACCAAACTCCAGCTGGCGCTTTGA
E)
In-frame start codons
CAAATGGC (307)
TTCATGTG (529)
CAGATGCA (583)
AACATGCA (868)
AACATGAC (883)
Intronic seq
Exonic seq
Kozak reliability
F)
WT1 isoforms
E7-GFP
P-GFP
DOX:
50kDa
40kDa
30kDa
25kDa
15kDa
80kDa
*
α-GFP
α-CNX
WT1 isoforms
E7-GFP
P-GFP
DOX:
50kDa
40kDa
30kDa
25kDa
*
α-WT1
G)
OVCAR3
TOV3291G
Log2 FC
CDH1
CDH2
FN1
SNAI1
SNAI2
TAGLN
ZEB1
ZEB2
H)
pvalue
start date for calculation
start date for calculation
pvalue
pvalue = 0.05
Min. survival curve median

Figure 7 supplemental: A) Left: 18s rRNA expression levels in human melanoma cell lines under normoxic conditions. Right: tWT1 expression levels in human melanoma cell lines presented as delta CT (dCT) to 18s under normoxic conditions. Statistical significance calculated by one-way ANOVA. *: $p < 0.05$; **: $p< 0.01$; ***: $p < 0.001$ **B)** tWT1 expression in ZR75 cells during the LTHY protocol in the presence of estradiol (E2), relative to the 5% O_2 timepoint. C**)** Top: Diagrams of the *WT1* locus in the human and murine genomes (not to scale). Middle and bottom: Respective tWt1 isoforms for each species with splicing patterns. Human tWT1 isoforms are G (ENST00000639907) and P (ENST00000652724). Purple: exon 1 for each transcript, derived from intron 5 of the canonical *WT1* locus. Orange: canonical WT1 exons. Pink: sequence introduced by a lack of splicing of isoform G intron 1. Green line: First in-frame ATG in each transcript. Diagrams not drawn to scale. **D)** Top: tWT1-G mRNA sequence from 5'UTR to end of CDS. Bottom: tWT1-P mRNA sequence from 5'UTR to end of CDS. Purple: Canonical WT1 intron 5 derived sequence. Orange: Canonical WT1 exon sequences. Red text: in-frame stop codons. White text: in-frame canonical start codons. Yellow text: alternative start codons. **E)** Kozak similarity scores for in-frame start codons in human P-tWT1. Purple: start codon is derived from canonical intronic sequences. Orange: Start codon is derived from exonic sequences. **F)** Western blot analysis of tWT1-GFP fusion constructs. E7-GFP was expressed in B16 cells, P-GFP was expressed in HEK cells. Membranes were probed with anti-GFP and anti-C-terminal WT1 to observe the various tWT1 constructs. Anti-calnexin was used as loading control. Expected protein is marked by asterisk. **G)** EMT-related gene expression signature in ovarian cancer cell lines at the end of the LTHY incubation. **H)** Two-sided p-value calculations from figure 7J given increasing start dates. P-value is calculated from the start date to the end of the dataset. Orange dashed line: pvalue = 0.05. Green dashed line: median of survival curves in figure 6F (1354 days). Right is a zoomed in view of the 1100-2000 day range.
